# Differential interactions of resting, activated, and desensitized states of the α7 nicotinic acetylcholine receptor with lipidic modulators

**DOI:** 10.1073/pnas.2208081119

**Published:** 2022-10-17

**Authors:** Yuxuan Zhuang, Colleen M. Noviello, Ryan E. Hibbs, Rebecca J. Howard, Erik Lindahl

**Affiliations:** ^a^Department of Biochemistry and Biophysics, Science for Life Laboratory, Stockholm University, PO Box 1031, Solna, 171 21 Sweden;; ^b^Department of Neuroscience, University of Texas Southwestern Medical Center, Dallas, TX 75390;; ^c^Department of Applied Physics, Swedish e-Science Research Center, KTH Royal Institute of Technology, PO Box 1031, Solna, 171 21 Sweden

**Keywords:** ligand-gated ion channel, nicotinic acetylcholine receptor, cholesterol, coarse-grained simulations, computational electrophysiology

## Abstract

The α7 nicotinic acetylcholine receptor is a pentameric ligand-gated ion channel that modulates neuronal excitability, largely by allowing Ca^2+^ permeation. Agonist binding promotes transition from a resting state to an activated state, and then rapidly to a desensitized state. Recently, cryogenic electron microscopy (cryo-EM) structures of the human α7 receptor in nanodiscs were reported in multiple conformations. These were selectively stabilized by inhibitory, activating, or potentiating compounds. However, the functional annotation of these structures and their differential interactions with unresolved lipids and ligands remain incomplete. Here, we characterized their ion permeation, membrane interactions, and ligand binding using computational electrophysiology, free-energy calculations, and coarse-grained molecular dynamics. In contrast to nonconductive structures in apparent resting and desensitized states, the structure determined in the presence of the potentiator PNU-120596 was consistent with an activated state permeable to Ca^2+^. Transition to this state was associated with compression and rearrangement of the membrane, particularly in the vicinity of the peripheral MX helix. An intersubunit transmembrane site was implicated in selective binding of either PNU-120596 in the activated state or cholesterol in the desensitized state. This substantiates functional assignment of all three lipid-embedded α7-receptor structures with ion-permeation simulations. It also proposes testable models of their state-dependent interactions with lipophilic ligands, including a mechanism for allosteric modulation at the transmembrane subunit interface.

## Significance

The α7 nicotinic acetylcholine receptor controls important electrical signaling processes in the nervous system, by allowing cations—particularly calcium—to cross membranes. Lipids and drugs are thought to regulate α7 by binding selectively to its resting, activated, or desensitized states. However, molecular details of these interactions are poorly understood, limiting prospects for drug design. We used molecular simulations to assign functional states to recent cryogenic electron microscopy structures and to quantify calcium permeation through the activated state, while cholesterol binds to a specific site in the desensitized state. Finally, we locate binding of the drug PNU-120596 (PNU) and propose a mechanism for α7 modulation in which PNU displacement of cholesterol promotes opening versus desensitizing.

Pentameric ligand-gated ion channels (pLGICs) are key mediators of electrochemical signal transduction in the nervous system (1). Upon the binding of neurotransmitters, the corresponding pLGICs open, allowing either anions or cations to cross the membrane for further signal transduction (2). The α7 nicotinic acetylcholine receptor (α7 nAChR), a subtype of the nicotinic superfamily of pLGICs, consists of five identical α7 subunits. It is an important part of the cholinergic nervous system; defects in this receptor are associated with neurological conditions including schizophrenia, Alzheimer’s disease, and autism spectrum disorders (3–5). The α7 nAChR is also widely expressed in the immune system and is linked to inflammatory disease (6, 7). Accordingly, this channel constitutes an important therapeutic target. Although no drugs in current practice specifically target the α7 nAChR (5), the type-II positive allosteric modulator PNU-120596 (PNU) has proved a valuable pharmacological tool (8–10). It prolongs channel opening and creates long-lived burst clusters in functional recordings. Binding sites for PNU have been proposed by both mutagenesis studies (11, 12) and molecular docking to homology models (13, 14) and were recently resolved in a detergent-embedded structure (15). However, we still lack comprehensive integration of functional, structural, and simulation data that explicitly links state-specific binding of PNU and other modulators to lipid interactions and channel conduction.

**Fig. 1.**
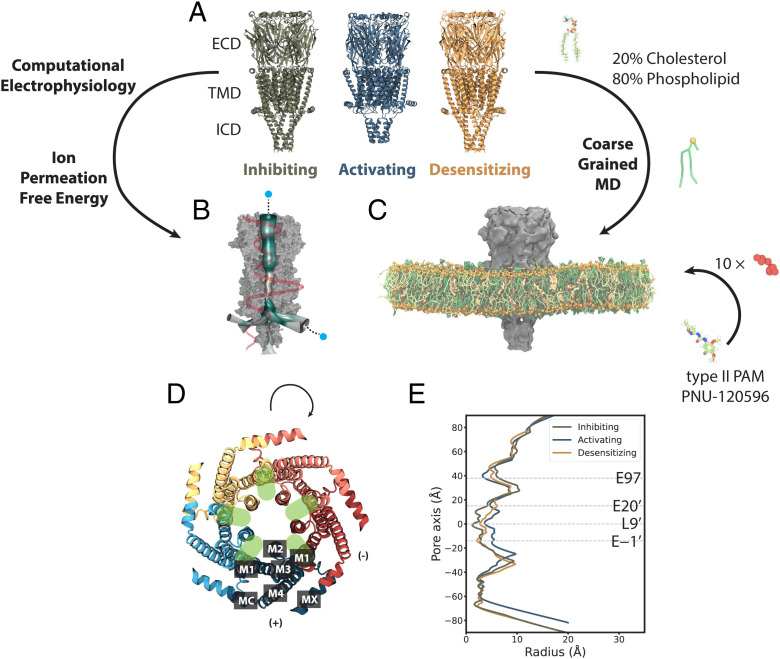
Computational approaches to test functional states and discover lipidic interactions of the human α7 nAChR. (*A*) Cryo-EM structures of the α7 nAChR in lipid nanodiscs, viewed from the membrane plane, determined in inhibiting (olive; PDB ID code 7KOO), activating (steel; PDB ID code 7KOX), and desensitizing (orange; PDB ID code 7KOQ) conditions, denoted as resting, activated, and desensitized states in this work, respectively. (*B*) Computational electrophysiology and ion-permeation free-energy profiles (orange curve) enable modeling of ion conductance and permeation. Mesh representation of the pore colored by hydrophobicity was generated by CHAP (72). (*C*) Coarse-grained simulations enable quantification of protein interactions with mixed-lipid membranes (80% phospholipids, green; 20% CHOL, yellow), as well as the ligand PNU (red). (*D*) Details of the α7-nAChR TMD, showing the structure under activating conditions (PDB ID code 7KOX) viewed from the extracellular side, colored by subunit. Labels on the bottom subunit indicate individual TMD helices M1 through M4, MX, and MA. Green ovals indicate intersubunit cavities proximal to the channel pore. (*E*) Pore-radius profiles [Rao et al. (72)] for the three experimental structures, colored as in *A*, with the midpoint (0 Å) of the channel axis at the 9′ hydrophobic gate. Dashed lines indicate key acidic residues, as well as the 9′ hydrophobic gate, facing the channel axis.

With the help of cryogenic electron microscopy (cryo-EM), three structures of the human α7 nAChR in lipid nanodiscs were recently reported in conditions expected to inhibit (bound to the antagonist α-bungarotoxin), activate (bound to the agonist epibatidine and modulator PNU), or desensitize (bound to only epibatidine) the receptor (16) (Fig. 1*A*). As for most pLGICs (17), the α7 nAChR can be dissected into an agonist-binding extracellular domain (ECD), a pore-forming transmembrane domain (TMD), and a semidisordered intracellular domain (ICD). All three domains are implicated in influencing ion permeation and selectivity (16, 18). The partially resolved ICD forms lateral portals thought to pass ions to the intracellular space, rather than the central vestibule (Fig. 1*B*) (18). Each subunit TMD consists of four membrane-spanning helices (M1 through M4) with the ring of M2 helices lining the central ion pathway of the pentamer (Fig. 1 *B* and *D*). The ECD contains a constriction at residue E97 (Fig. 1*E*) and has a Ca^2+^ binding site (16, 19) alongside the permeation pathway near the ECD–TMD junction.

The α7 lipid-nanodisc structure obtained under activating conditions is strikingly different from those in either inhibiting or desensitizing conditions. In particular, the TMD helices are tilted and translated (Fig. 1*A*), the surrounding membrane appears to be compressed, and the M4–MA helix partially loses its secondary structure. It remains to be determined whether this unanticipated state could account for distinctive permeation properties of the α7 subtype, e.g., relative selectivity for Ca^2+^ ions (16, 20) and fast desensitization (21). Furthermore, comparing α7-nAChR structures highlights potentially important state-dependent interactions with the membrane. Membrane components, including cholesterol (CHOL), have been shown to modulate nAChRs by binding directly to the TMD, as well as altering bulk lipid properties (22–24). Indeed, specific lipid interactions may critically influence the function of several pLGICs, especially in eukaryotes (25). In the case of α7, no specific lipids were resolved, leaving open questions as to the molecular details of state-specific remodeling at the protein–lipid interface.

Although the presumed activating conditions included a saturating concentration of PNU, this modulator could not be confidently resolved anywhere around the protein from the electron density. During preparation of this manuscript, a second set (15) of α7-nAChR structures were reported under presumed resting (apo), partially desensitizing (agonist EVP-612+PNU–bound), and desensitizing (only EVP-612–bound) conditions. Although these were determined in detergent micelles, the structures in resting and desensitizing conditions were notably similar to those previously reported in lipid nanodiscs in inhibiting and desensitizing conditions, respectively. The PNU-bound structures were more divergent, with the detergent complex containing a narrower pore, less likely to represent a fully activated state. Moreover, PNU densities resolved in the intersubunit cavities of the detergent structure could not be superimposed in the lipid-nanodisc structure without steric clashes. Thus, the location and mechanism of PNU potentiation in the activated state remain unclear.

Molecular dynamics (MD) simulations complement experimental tools and have made it possible to study both ion permeation (26) and lipid/drug interactions of ion channels (27, 28). Here, we report an MD study of three lipid-embedded experimental structures of the α7 nAChR, covering three key functional states. We tested the permeation properties of the structure under activating conditions, utilizing both computational electrophysiology and ion-permeation calculations, in comparison to experimental conductance and selectivity. We then quantified interactions of lipid molecules and PNU with different states of the channel, using coarse-grained simulations that make it possible to reach timescales where lipids diffuse to preferentially interact with different parts of the membrane protein. Our results detail lipidic interactions of specific residues in multiple TMD regions and substantiate a mechanism of PNU–CHOL dynamics that may underlie modulation of the α7-nAChR gating cycle.

## Results

### An Open Functional State under Activating Conditions.

**Fig. 2.**
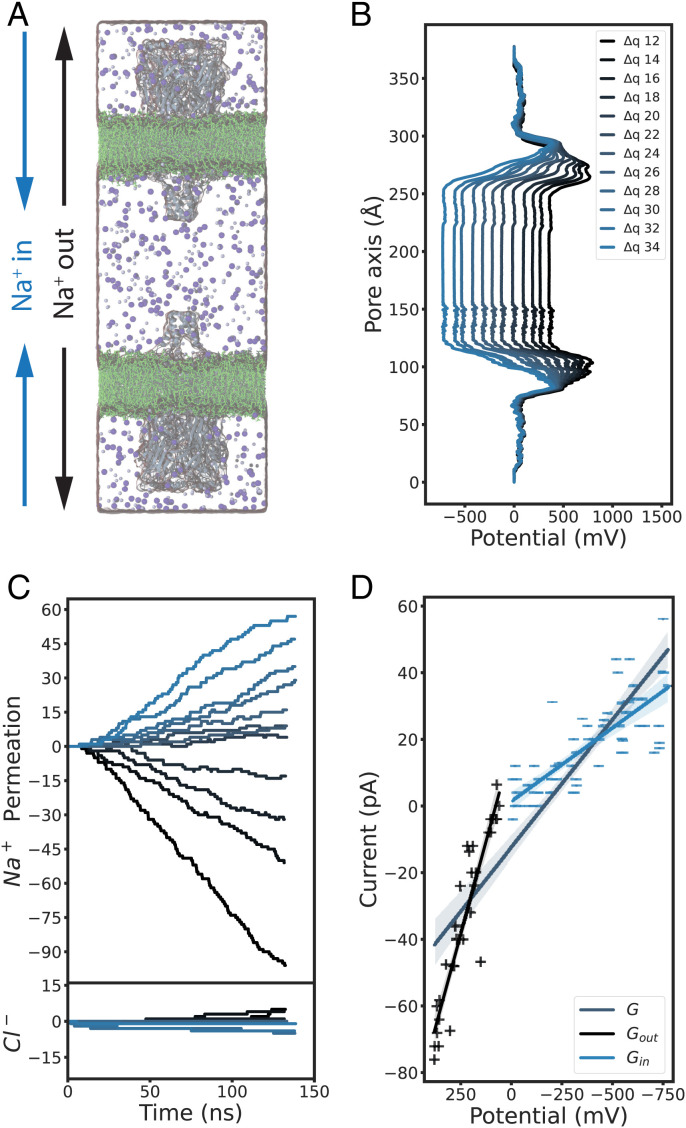
A conducting functional state under activating conditions. (*A*) Snapshot of a computational electrophysiology simulation, with 150 mM NaCl (Na^+^, blue; Cl^−^, purple) surrounding two α7 nAChRs (gray) embedded in separate membrane patches (green) in an antiparallel setup. (*B*) Averaged electrostatic potential profiles along the membrane normal as shown in *A*, with various ion imbalances (differential of 12 to 34 charges, navy to light blue) between the two water compartments separated by the membranes. (*C*) Permeation events over time for Na^+^ (*Upper*) or Cl^−^ ions (*Lower*) in individual computational electrophysiology simulations conducted at membrane potentials, colored as in *B* (depolarized to polarized conditions, navy to light blue). Positive permeation events refer to ions passing from outer to inner compartments (“Na^+^ in,” *A*); negative values refer to ions passing the opposite direction. (*D*) Current–voltage plot derived from computational electrophysiology simulations, as shown in *C*. Solid lines show linear fits (with 95% CI) to currents measured at depolarized potentials (navy +; *G*_out_ = 223.0 ± 16.5 pS), currents at polarized potentials (light blue −; *G*_in_ = 44.2 ± 4.4 pS), or the full dataset (steel; *G*_full_ = 76.6 ± 4.1 pS).

To test the functional state of the α7-nAChR structure determined under activating conditions, we first examined its ion conductance using computational electrophysiology (29, 30). Two copies of the cryo-EM structure (Protein Data Bank [PDB] ID code 7KOX; determined in the presence of epibatidine and PNU) were embedded in lipid bilayers oriented opposite one another in a single simulation box, enabling the generation of a range of electrostatic potentials in an ∼150 mM NaCl medium (Fig. 2*A*). In the presence of voltage differences from −700 mV to 400 mV (Fig. 2*B*), Na^+^ preferably permeated the channel, with a selectivity of ∼10:1 over Cl^−^ (Fig. 2*C*). From the slope of the current–voltage relationship, Na^+^ conductance was estimated at *G* = 76.6 ± 4.1 pS, consistent with an open, cation-selective state.

Interestingly, Na^+^ current–voltage relationships at polarized potentials fit a shallower conductance slope (*G*_in_ = 44.2 ± 4.4 pS) than those at depolarized potentials (*G*_out_ = 223.0 ± 16.5 pS) (Fig. 2*D*). Indeed, the estimated depolarized (outward) Na^+^ conductance was remarkably similar to experimentally measured 192 pS (16). This preference could reflect a modest outward rectification, or it could be a consequence of model or parameter bias, possibly underestimating inward flux. To test the robustness and determinants of this effect, we ran additional simulations of a single receptor in the presence of a hyperpolarized (−200 mV) or depolarized (+200 mV) external electric field. As expected, the structure determined under desensitizing conditions (PDB ID code 7KOQ; with epibatidine alone) was effectively nonconductive in both conditions. Conversely, the structure under activating conditions exhibited comparable Na^+^ conductance as in our computational electrophysiology simulations, with lower inward than outward values (*SI Appendix*, Fig. S1*A*).

Notably, deletion of the ECD produced a partial receptor with elevated conductance in both directions and abolished the preference for outward flux, suggesting that this domain limits Na^+^ permeation particularly in the inward direction. In contrast, deletion of the ICD bundles resulted in conductance values comparable to wild type in both directions, substantiating the importance of the ECD in suppressing Na^+^ efflux. Removing the sidechain at the tightest constriction in the ECD vestibule (E97A) also failed to relieve the apparent inhibition in either direction, indicating that conductance determinants are located elsewhere. A possible contributing factor was the presence of five Ca^2+^ ions, which were resolved at the ECD–TMD interface (16, 19) and, accordingly, included in our computational electrophysiology experiments, as well as permeation calculations described below. In applied-field simulations, Ca^2+^ ions spontaneously displaced Na^+^ to enter this site (*SI Appendix*, Fig. S2 *A* and *B*) and remained stably bound when equilibrated at their structurally resolved positions (*SI Appendix*, Fig. S2*C*). Coordinated by acidic residues D41, D43, E44, and E172, these ions were distal to the conduction pathway, but raised the effective potential, particularly in the outer half of the TMD pore (*SI Appendix*, Fig. S3). However, including these bound ions in our applied-field simulations only slightly elevated the conductance in both directions and did not substantially alter the preference (*SI Appendix*, Fig. S1*A*), suggesting that local ion interactions in this region contribute little to the apparent ECD barrier to ion flow. Including a higher concentration of bulk Ca^2+^ ions, equivalent to that of Na^+^ (150 mM each), resulted in a roughly 1:1 permeation ratio—i.e., ∼2-fold greater conductance for Ca^2+^ versus Na^+^ ions, as expected for a divalent cation of equivalent permeability (*SI Appendix*, Fig. S1*B*).

### State-Dependent Ion Interactions from Permeation Free-Energy Profiles.

**Fig. 3.**
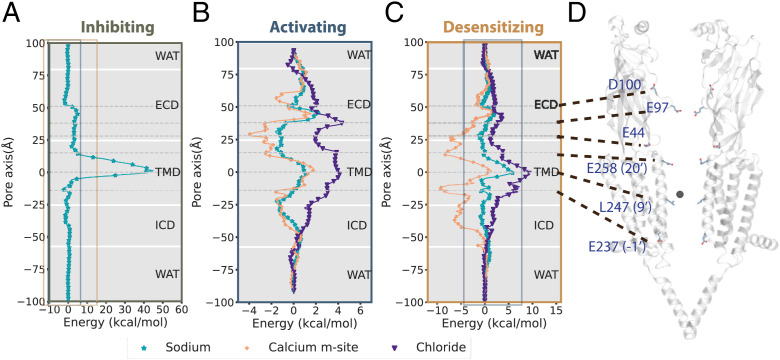
State-dependent ion interactions from permeation free-energy profiles. (*A*) Free-energy profile of Na^+^ ion permeation (teal) through the lipid-embedded α7-nAChR structure under inhibiting conditions (PDB ID code 7KOO). Dashed lines indicate key acidic residues, as well as the 9′ hydrophobic gate, facing the channel axis in the ECD and TMD. Solid boxes indicate zoom windows depicted in *B* (steel) and *C* (orange). (*B*) Free-energy profiles of Na^+^ (teal), Ca^2+^ (ochre), and Cl^−^ (indigo) permeation through the structure under activating conditions (PDB ID code 7KOX), with key residues indicated as in *A*. All energy barriers are substantially reduced relative to inhibiting and desensitizing conditions, with the lowest barriers for Ca^2+^. (*C*) Free-energy profiles of ion permeation through the structure under desensitizing conditions (PDB ID code 7KOQ), colored as in *B*, with key residues indicated as in *A*. The solid steel box indicates the zoom window depicted in *B*. (*D*) Model of the structure under activating conditions; for clarity, only two opposing subunits are shown. Key residues labeled in *A*–*C* are shown as sticks.

To further characterize the functional states and ion interactions of each lipid-embedded cryo-EM structure, we next calculated permeation free-energy profiles for various ions and states, using the accelerated weight histogram (AWH) method (31, 32). This approach to enhanced sampling is particularly suited to modeling the nonlinear conduction pathway of nAChRs, in which ions are expected to permeate the fenestrations between ICD helices (Fig. 1*B* and *SI Appendix*, Fig. S2*A*). The structure in the presence of inhibitory α-bungarotoxin (PDB ID code 7KOO) featured a 40 kcal/mol free-energy barrier at the midpoint of the TMD. This apparent gate was centered at L247 (9′) (Fig. 3*A*), a conserved residue known to form a hydrophobic gate in resting pLGICs (33, 34). We henceforth considered this structure in the resting state. In contrast, under activating conditions (PDB ID code 7KOX), the 9′ barrier decreased below 4 kcal/mol for Na^+^, K^+^, or Ca^2+^, comparable to a low barrier at the outer end of the ECD (Fig. 3*B*). Thus, based on its behavior in computational electrophysiology, applied-field, and ion-permeation simulations, this activated-state structure was apparently open.

The α7 nAChR is known to be selective for cations (35). Indeed, Cl^−^ interactions were unfavorable throughout the activated-state conduction pathway and particularly elevated relative to positive charges at 9′ and at the ring of E97 sidechains forming the tightest ECD constriction (Fig. 3*B*). Conversely, favorable energy wells were observed for cations both at the outer end of the ECD near D100 and at the TMD–ICD interface (Fig. 3*B*). Notably, cations were directly coordinated by protein oxygen atoms at several positions in the ECD and at the outer and inner ends of the TMD pore (*SI Appendix*, Fig. S4 *A* and *B*), possibly contributing to selectivity. As α7 nAChRs are particularly permeable to Ca^2+^ compared to other subtypes (20), we sought to model Ca^2+^ interactions as accurately as possible, adopting recent parameters shown to be more accurate than default values in CHARMM36 (36). Consistent with previous reports, we found this model to reasonably represent Ca^2+^ hydration (*SI Appendix*, Fig. S5) and to relieve potentially overestimated protein–Ca^2+^ interactions, particularly at the intracellular mouth of the pore (*SI Appendix*, Fig. S6*D*). In our calculations, aside from the peripheral binding site (near E44; *SI Appendix*, Fig. S7*B*), Ca^2+^ made several favorable interactions along the conduction pathway, including energy wells below −2 kcal/mol at the E97 constriction in the ECD, E258 (20′) in the outer TMD, and E237 (−1′) at the inner mouth of the TMD pore. These minima were consistent with ion densities in our unbiased simulations with an applied electric field (*SI Appendix*, Fig. S2*A*). Indeed, similar free-energy barriers were calculated for Ca^2+^ and Na^+^ (Fig. 3*B*), possibly corresponding to the ∼1:1 selectivity observed in the presence of an equal ion ratio in electric-field simulations (*SI Appendix*, Fig. S1*B*).

Consistent with our free-energy profiles, experimental structures were reported with five Ca^2+^ ions peripheral to the ion-permeation pathway (*SI Appendix*, Fig. S7*B*), near the five symmetric E44 residues. To test the influence of bound Ca^2+^ on ion permeation, we also ran free-energy calculations with Ca^2+^ at the five E44 sites in the structure determined under activating conditions (*SI Appendix*, Fig. S7). The presence of Ca^2+^ had no effect on the profile for Cl^−^ permeation. For Ca^2+^, it relieved the free-energy well for further Ca^2+^ interactions at E44, but it did not substantially alter the permeation landscape elsewhere. Bound Ca^2+^ elevated the free-energy barrier for Na^+^ more broadly across the ECD–TMD interface, between E97 and E237, although the predominant barrier remained at the outer ECD around D100. Thus, inclusion of Ca^2+^ did not qualitatively alter the apparent permeation or selectivity of this structure, though local effects on ECD dynamics in the vicinity of E44 remain to be explored.

Under desensitizing conditions, the principal barrier to ion conduction was, again, found at the 9′ hydrophobic gate, and it was elevated relative to the activated state (Fig. 3*C* and *SI Appendix*, Fig. S6*C*). Notably, monovalent cations had to release at least one hydration water molecule in order to transit the 9′ gate in this structure, while in the activated state, they retained a full hydration shell (*SI Appendix*, Fig. S4 *A*, *C*, and *E*). Ion-coordinating waters were further substituted by protein oxygen atoms at the inner mouth of the pore (E237, −1′; *SI Appendix*, Fig. S4 *E* and *F*), though this position did not constitute a substantial free-energy barrier (Fig. 3*C*). For Ca^2+^, several free-energy wells were especially pronounced under desensitizing conditions, with interaction energies below −7 kcal/mol at E44, E258 (20′), and E237 (−1′) relative to bulk solvent (Fig. 3*C*). Accordingly, while Ca^2+^ ions were bound even more strongly in the pore of this structure than in the activated state, they also faced a more substantial (9 kcal/mol) barrier to transit the hydrophobic gate. Thus, along with the lack of conduction observed for this structure in applied-field simulations (*SI Appendix*, Fig. S1*A*), we henceforth considered it a plausible desensitized state.

### State-Dependent Lipid Interactions.

With functional states assigned to each of the α7 nicotinic receptor structures, we further investigated lipid interactions in each state. Compared to the apparent resting and desensitized states, the activated-state experimental structure featured a distinctive translocation of the MX helix toward the membrane core (Fig. 1*A*). This correlated to a compression of lipid density in the vicinity of the protein (16). The timescales of lipid diffusion and remodeling in mixed membranes are typically prohibitive to all-atom simulations. Therefore, to test the extent and implications of this apparent membrane compression, we applied coarse-grained simulations to each structure modeled with Martini 2.2 (37). To approximate the experimental system as closely as possible, each structure was embedded in a mixed bilayer with 20% CHOL and simulated with protein backbone restraints for 20 µs (*Methods*). We then analyzed protein–lipid interactions in the equilibrated systems during the final 5 µs of simulation time.

**Fig. 4.**
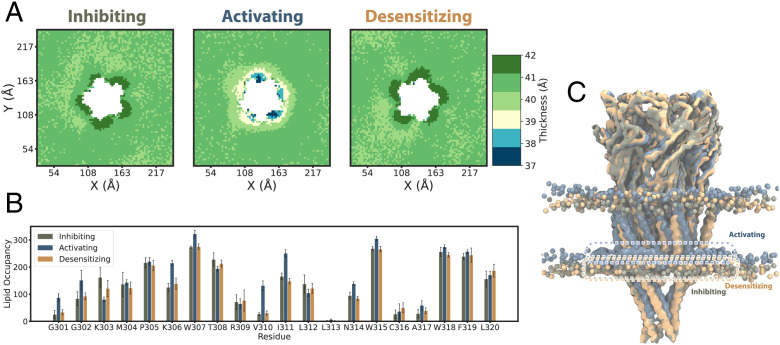
Local membrane compression in the activated state, demonstrated by coarse-grained simulations. (*A*) Membrane thickness, colored according to the scale bar at the far right, averaged over the last 5 μs from simulations of the lipid-embedded α7-nAChR structure under inhibiting (resting), activating (activated; *Center*), or desensitizing (desensitized; *Right*) conditions. The membrane thickness immediately proximal to the protein was comparable in the apparent resting and desensitized states (∼42 Å), while it was compressed in the activated state (37 to 39 Å). (*B*) Occupancy of lipid interactions at each residue of the MX helix, showing increased contacts at several residues in the activated state (gray). (*C*) Overlay of the last snapshots from simulations of the apparent resting (olive), activated (gray), and desensitized (orange) states, aligned on the ECD. Lipids of the inner leaflet are relatively displaced toward the membrane core by the distinct conformation of the activated state. Dashed boxes indicate positions of the membrane-peripheral MX helix in each structure. Only the membrane within 60 Å of the protein is shown for clarity.

Membrane thickness proximal to the protein was strikingly state-dependent (Fig. 4*A*). Whereas the resting and desensitized states were embedded in comparable local environments up to 42 Å thick, the activated state compressed the surrounding membrane to as little as 37 Å. After 20-µs coarse-grained simulation of the open structure, lipid heads from the inner leaflet translocated “up” toward the bilayer core (Fig. 4*C*), increasing their interactions with the MX helix relative to the resting and desensitized states (Fig. 4*B*). This effect dissipated within 60 Å from the protein center (*SI Appendix*, Fig. S8), restoring the bulk membrane to roughly 40-Å thickness (Fig. 4*A* and *SI Appendix*, Fig. S8). The free-energy cost for this compression was estimated to be 0.7 or 0.4 kcal/mol for the resting-to-activated or desensitized-to-activated transitions, respectively (*Methods*).

**Fig. 5.**
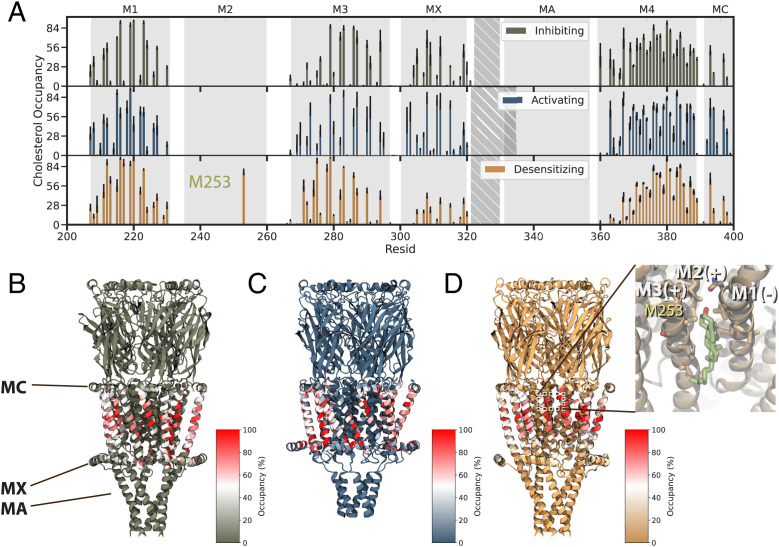
Altered CHOL interactions in the desensitized state. (*A*) Percent occupancy of CHOL interactions with each residue of the α7 nAChR in 20-μs coarse-grained simulations of structures determined under inhibiting (*Top*), activating (*Middle*), and desensitizing (*Bottom*) conditions. Whereas inhibiting and activating conditions were associated with comparable CHOL interactions in the transmembrane core and MX helix, desensitizing conditions preferred interactions in the outer leaflet, including M2 residue M253. (*B*) CHOL occupancies as in *A,* colored according to the scale bar and mapped onto the experimental structure under inhibiting conditions, with key membrane-facing or peripheral helices labeled. (*C*) CHOL occupancies as in *B* for the structure under activating conditions. (*D*) CHOL occupancies as in *B* for the structure under desensitizing conditions. *D*, *Inset* shows CHOL (green) and associated residues backmapped to atomic coordinates, including M253 from the principal M2 helix, in the upper-leaflet site preferred in this state.

Aside from the differences in bulk membrane properties described above, the coarse-grained simulations of α7-nAChR structures also indicated state-dependent interactions with specific lipids. Notably, CHOL has been shown to modulate desensitization in nAChRs, although its role in the α7 subtype is unclear (Rankin et al., 1997 (38) ). In simulations of the resting and activated states, CHOL interacted with membrane-facing residues in the M1, M3, M4, and MX helices (Fig. 5 *A*–*C* and *SI Appendix*, Fig. S9), consistent with densities attributed to CHOL in other recent α7 structures (*SI Appendix*, Fig. S10) [Zhao et al. (15)]. In simulations of the desensitized state, CHOL contacts shifted away from MX toward the outer-leaflet faces of M1 and M3. Moreover, CHOL interacted extensively (>75% occupancy) with residue M253 (15′) in the pore-lining M2 helix in the desensitized state, via a cavity at the subunit interface (Fig. 5 *A* and *D*). This interaction was not observed in corresponding resting- or activated-state structures, consistent with a role for intersubunit CHOL binding in facilitating the distinctive desensitization profile of α7 nAChRs. Interestingly, this site overlapped with PNU density inside a partially desensitized/activated state of the α7 receptor in detergent (15). These findings led us to further investigate possible PNU binding sites in the fully activated state.

### Allosteric Potentiator PNU Preferentially Occupies an Intersubunit Site in the Activated State.

The presumed activated state was resolved in the presence of lipids with both the agonist epibatidine and positive allosteric modulator PNU (16). However, despite saturating concentrations (200 μM), no PNU molecules could be definitively built in the cryo-EM density. To elucidate the binding mode of this modulator, we ran additional coarse-grained simulations of each experimental structure in the presence of PNU. Each system was built with 10 PNU molecules placed randomly at a distance of 20 Å from the protein surface and simulated in quadruplicate for 20 μs using Martini 3 (39, 40). This force field, recently reported to be better optimized for protein–ligand interactions (40), recapitulated PNU properties optimized with quantum-mechanical or atomistic approaches beforehand (*SI Appendix*, Fig. S11).

**Fig. 6.**
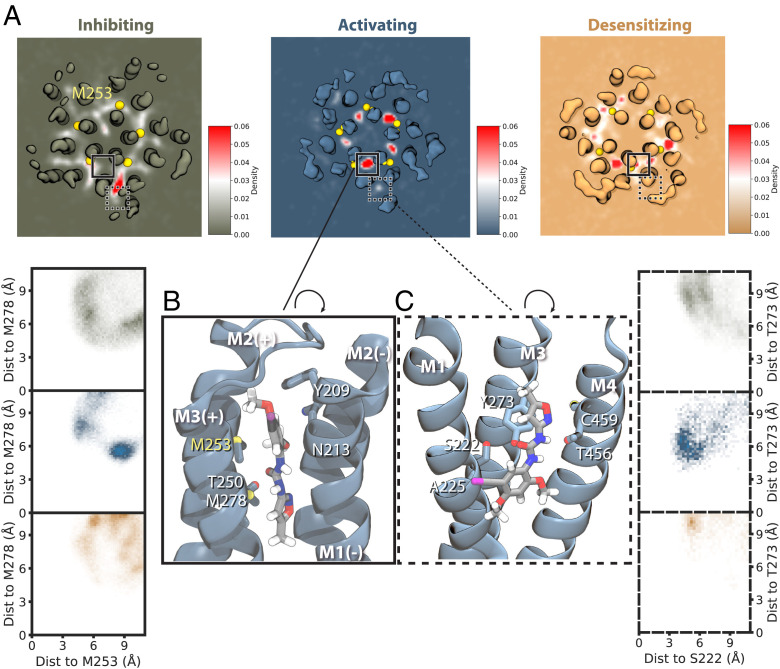
Allosteric potentiator PNU preferentially occupies an intersubunit site in the activated state. (*A*) Average PNU density maps derived from quadruplicate 20-μs coarse-grained simulations, shown for a representative sliced through the TMD (*SI Appendix*, Fig. S12). A primary, intersubunit and secondary, peripheral site for PNU in the activated state are indicated by solid and dashed boxes, respectively. The intersubunit density of PNU was observed with relative symmetry over all five interfaces in the activated state (blue). (*B*) PNU binding in the intersubunit site, backmapped to an all-atom model. *B*, *Inset* plots the two-dimensional density distributions of PNU based on its distance (Dist) to M278 (*y* axis) and M253 (*x* axis). It shows preferential occupation of a site bounded by these residues in the activated state (*Middle*), relative to more diffuse distributions in resting (*Top*) and desensitized (*Bottom*) states. (*C*) Backmapped representation of PNU binding in the peripheral site, with *Inset* as in *B* showing occupation of a pose bounded by residues T273 (*y* axis) and S222 (*x* axis) in the activated state (*Middle*), relative to more distant/diffuse distributions in resting (*Top*) and desensitized (*Bottom*) states.

Although average PNU densities were relatively diffuse and bound asymmetrically across the five subunits in simulations of the resting or desensitized states (Fig. 6*A*), they consistently occupied sites in all five subunits facing the outer leaflet in the activated state symmetrically. Each of five primary, intersubunit outer-leaflet sites was bounded by the M2 and M3 helices of the principal subunit and M1 and M2 of the complementary subunit (Fig. 6*B*). Specifically, PNU bound to the activated-state structure preferentially assumed a pose 8 Å from M2 residue M253 and 5 Å from M3 residue M278 (center-of-mass distance), whereas interactions in other states sampled a wider range of poses. A secondary, peripheral outer-leaflet site was bounded by the M1, M3, and M4 helices of each individual subunit (Fig. 6 *A* and *C*). Although this site was visited with lower occupancy than the intersubunit site, PNU appeared to prefer an associated pose 5 Å from M1 residue S222 and 6 Å from M3 residue T273 in the activated state, in contrast to more distant and diffuse interactions in other states (Fig. 6*C*). Mutations near both the intersubunit (M253L) and peripheral (S222M and C459Y) PNU sites were previously shown to disrupt PNU potentiation (11, 13, 41), indicating that both may contribute to binding of this modulator.

Interestingly, recent structures of the α7 nAChR in detergent also showed PNU at an intersubunit outer-leaflet site (15). Although the PNU site in this state, described as partially desensitized/activated, involved similar key residues as in our simulations, substantial differences in the helical backbone render them partially incompatible. If aligned on the M2 helices, the resolved pose for PNU—with its long axis parallel to the membrane plane—would clash with the outer M3 helix in our simulations (*SI Appendix*, Fig. S12), indicating that the primary pose described here is specific to the more expanded lipid-bound activated state.

To further test the role of the primary, intersubunit site in PNU binding, we ran additional coarse-grained PNU simulations of the activated structure with the mutation M253L. Despite the relatively conservative nature of this substitution, it consistently disrupted occupancy at the intersubunit site relative to wild type (*SI Appendix*, Fig. S13). In M253L simulations, PNU instead occupied a novel inner-leaflet site, including contacts with M1 residue A225 (*SI Appendix*, Fig. S13). Interestingly, the substitution A225D was also previously shown to disrupt PNU potentiation (13). In simulations of this A225D mutant, PNU again occupied the inner-leaflet site, making frequent interactions between its polar ureido group and the introduced aspartate at position 225 (*SI Appendix*, Fig. S13*C*). A double mutant containing A225D and M253L exhibited a similar PNU as in both single mutants (*SI Appendix*, Fig. S13), consistent with a common structural effect of either disruption in the primary upper-leaflet site or enhanced binding in the lower-leaflet site.

Notably, our coarse-grained simulations indicated a pore-mediated pathway of PNU transit between intersubunit sites (*SI Appendix*, Fig. S14 and Movie S15). After entering an intersubunit site via the membrane, a PNU molecule could spontaneously transit the interface between M2 helices to enter the pore and then enter an equivalent site at another subunit interface. To partially quantify this phenomenon, we backmapped the activated-state coarse-grained system to atomistic resolution and used umbrella sampling to characterize this pore-mediated pathway. As expected, we found one free-energy minimum within each intersubunit site and a second in the open pore (*SI Appendix*, Fig. S14*B*). A capacity for dynamic exchange between intersubunit sites could contribute to poor PNU resolution, even in the context of functionally relevant binding.

## Discussion

Understanding the gating cycle of ligand-gated ion channels requires comprehensive atomic details of functional endpoints, but also correlations with functional data and details of interactions with modulators and the lipid-membrane components. With improvements in single-particle cryo-EM techniques, several pLGIC structures have now been resolved in the context of various ligands and lipidic environments. However, there are only a few cases in which a single construct has been reported in resting, activated, and desensitized states. In this context, the recent report of structures of the lipid-embedded α7 nAChR under inhibiting, activating, and desensitizing conditions provides an invaluable opportunity to mechanistic and dynamic modeling, not least due to the novel free-energy profile and properties of the proposed activated state. A particularly critical challenge is the accurate assignment of functional states to experimental structures—for example, by the application of MD simulations of ion and lipid interactions, which we have pursued by combining electrostatic pore profiling, computational electrophysiology, applied electric fields, and enhanced sampling of ion permeation.

For the α7 nAChR, we found that simulations of the structure determined in presumed activating conditions—in the presence of both agonist (epibatidine) and potentiator (PNU)—produced single-channel conductance comparable to laboratory electrophysiology experiments (16), which confirms its assignment as a plausible activated state. This assignment was further validated by simulations in the presence of an applied electrical field and by the low barrier to cation permeation, as determined by enhanced sampling. In contrast, the inhibited structure—determined with α-bungarotoxin—could be assigned to a resting state in simulations, with a major barrier to permeation at the central hydrophobic gate characteristic of these channels. A third structure, stably bound to an agonist (epibatidine), but impermeable to ions in our simulations, was accordingly assigned to a desensitized state. Interestingly, the predominant barrier to ion conduction in this structure remained at the central gate, albeit to a lesser extent than in the resting state. This free-energy profile contrasted with those of desensitized gamma-aminobutyric acid-type A receptors, among others, in which the predominant gate was shown to shift to the intracellular end of the pore (42). It remains to be determined whether this apparently distinct desensitization profile is shared by other nAChRs or in the larger pLGIC family.

Surprisingly, our simulations indicated a higher outward than inward conductance for Na^+^ ions in the activated state. In contrast, previous experimental work suggested a moderate inward rectification for α7 nAChRs (43, 44). However, given that our simulations were performed in idealized computational conditions—lacking, for example, intracellular polyamines (45) and substantial regions of the ICD—it is not trivial to assess the biophysical or physiological relevance of this behavior. Simulations in the presence of an electric field indicated that the apparent outward rectification could be attributed to differential inhibitory interactions in the ECD, without which conductance was enhanced to a similar level in both directions. A similar effect of the ECD in limiting cation conductance was previously observed in the bacterial homolog GLIC (46), indicating a complex role of this domain in regulating permeation, as well as agonist binding. The outward rectification effect was not substantially modified by the presence or absence of the ICD, nor by an extracellular ring of glutamate residues (E97) at the tightest constriction in the channel pathway. An apparently structural set of modulatory Ca^2+^ ions, located peripheral to the conduction pathway in the ECD, also had little effect on conduction, though they remained stably bound throughout our simulations (*SI Appendix*, Fig. S3*C*).

Both our computational electrophysiology and ion-permeation calculations demonstrated selectivity for cations over anions in the activated state. Notably, the free-energy barrier and selectivity for Ca^2+^ permeation was comparable to that for Na^+^, particularly with Ca^2+^ bound at its structurally resolved sites. Accordingly, these channels should import substantial Ca^2+^ under physiological conditions, where intracellular buffering elevates the driving force relative to other cations. Using a recently refined Ca^2+^ model shown to approach the performance of some polarizable force fields (36), we were able to identify local interaction sites for Ca^2+^ along the permeation pathway, including acidic residues E97 and E258, previously shown to influence Ca^2+^ selectivity (16). Moreover, with Ca^2+^ bound at its resolved sites in the ECD, interaction energies for Na^+^ were moderately elevated around the ECD–TMD interface. Meanwhile, Ca^2+^ interactions were more favorable relative to bulk solvent. Including the five structural Ca^2+^ ions in our simulation box approximated physiological extracellular concentrations of ∼2 mM, indicating that these preferences approximate a physiological profile. Abolishing Ca^2+^ binding at this site by the mutation D43A has been shown to decrease Ca^2+^ selectivity in electrophysiology experiments (47). It is consistent with a role for these bound ions in promoting Ca^2+^ permeation, possibly by a charge/space competition mechanism (48, 49).

Coarse-grained simulations provided insights into the longer-timescale dynamics of lipid interactions and channel rearrangements associated with gating. Most dramatically, the activated state was associated with local compression of the membrane relative to resting or desensitized states and increased interactions of the intracellular MX helices with membrane lipids. It is interesting to consider whether MX–lipid interactions may contribute to alleviating the free-energy penalty of compressing the lipid bilayer. A similar relative motion of the MX helices toward the TMD has been reported in 5-HT_3_ receptors (50, 51), indicating that such a conformational shift could be linked to a conserved mechanism of activation. Conversely, the desensitized state was associated with differential CHOL interactions compared to resting or activated states, providing testable hypotheses for future work to refine our understanding of the established CHOL dependence of gating in these channels (22–24). Moreover, we were able to apply the improved small-molecule interaction properties of the Martini 3 force field (39, 40) to identify the activated-state binding pose of PNU, which was present, but not resolvable, in the lipid-nanodisc cryo-EM structure. In our simulations, PNU spontaneously transitioned between equivalent binding sites at the five subunit interfaces, suggesting dynamic behavior that could contribute to relatively poor resolution of the average experimental electron density. Interestingly, PNU occupied a similar region, but in a substantially different orientation, in a proposed intermediate state of the α7 receptor reported in detergent after initiation of this simulation work. Coarse-grained simulations may prove similarly valuable in characterizing state-dependent interactions, particularly of lipophilic modulators, as alternative structural models become increasingly available.

**Fig. 7.**
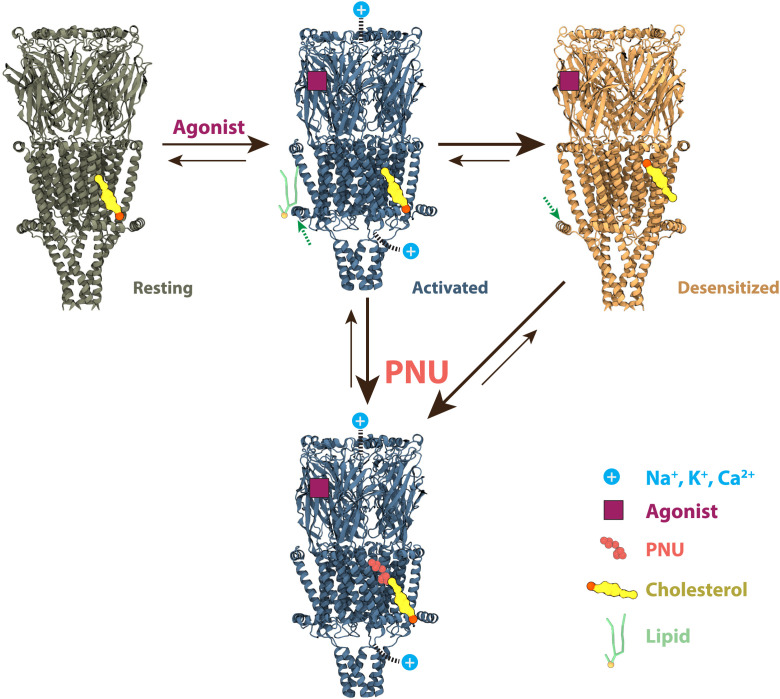
Proposed mechanism for α7 gating in the presence of agonist, with or without PNU. Starting from a resting state (olive), binding of agonist (purple) promotes transition to a Ca^2+^-permeable activated state (blue; *Upper*) with local membrane compression and increased contacts of the MX helix with lipids (green). This state can be relatively stabilized by binding of PNU (salmon) to a site near the middle of the TMD (blue; *Lower*). In the absence of PNU, the activated state transitions rapidly to a desensitized state (orange), with CHOL (yellow) relocated to the middle-TMD site.

Taken together, the atomic-resolution and coarse-grained simulations in this work support a structurally detailed mechanism for a basic three-state gating cycle in receptors (Fig. 7). Binding of the agonist in the ECD promotes opening of a cation-permeable, and specifically Ca^2+^-permeable, pore, accompanied by local compression of the bilayer and increased contacts of the MX helices with the lower membrane leaflet. With continued agonist exposure, the channel would be expected to transition rapidly to a desensitized state, with a partially contracted pore and a shift in preferential CHOL binding from a lower- to middle-leaflet site. However, binding of PNU—possibly in competition for this middle-leaflet site—relatively stabilizes the activated state, opposing desensitization (*SI Appendix*, Fig. S16). Although further structure–function work may refine this mechanism, including the likely contribution of additional resting, activated, desensitized, and intermediate states, this mechanism is consistent with the broad functional properties of the α7 receptors and illustrates the evolving utility of MD simulations in annotating and interpreting structural data for pharmacologically important systems.

## Methods

### General All-Atom MD-Simulation Setup.

Coordinates for α7-receptor cryo-EM structures determined under apparent inhibiting, activating, and desensitizing conditions (PDB ID codes 7KOO, 7KOX, and 7KOQ, respectively) were used as starting models for all simulations. Where present, the α-bungarotoxin, epibatidine, and Ca^2+^ ions were placed as in the deposited structures. The CHARMM36m (52) (July 2020 version) force field was used to describe each protein, which was embedded in a bilayer of 400 1-palmitoyl-2-oleoyl-*sn*-glycero-3-phosphocholine (POPC) molecules and solvated in a cubic box by using CHARMM-GUI (53, 54). The TIP3P (55) water model and NaCl were added to bring the system to neutral charge and an ionic strength of 0.15 M. In total, 212 chloride ions, 242 sodium ions, and 76,871 water molecules were added into a 12.8-nm × 12.8-nm × 18.6-nm simulation box.

Atomistic simulations were performed with either default Ca^2+^ parameters or recently revised versions (36), as indicated. Small-molecule parameters for epibatidine and PNU were generated with CGenFF (56) and optimized with FFParam (57) together with Psi4 (58) as the quantum chemistry backend. The modified version of the forcefield (59) can be found as indicated in *Data, Materials, and Software Availability*.

All simulations were performed with GROMACS 2020 (60). Default settings of the CHARMM36 force field (which can be found in the trajectory/mdp folder in the Zenodo repository (59) provided in *Data, Materials, and Software Availability*) were applied during energy minimization and equilibration. Each system was energy-minimized, then equilibrated for 250 ps with a constant number of particles, volume, and temperature when both protein and lipid molecules were restrained. Each system was then equilibrated with a constant number of particles, pressure, and temperature for 40 ns, during which the position restraints on the protein were gradually released. Weak thermostats and barostats (61) were used to model the system at 300 K and 1 bar during relaxation, and bond lengths were constrained by using the LINCS (62) algorithm.

### Computational Electrophysiology.

The equilibrated simulation box was used as the starting configuration. A second box was then translated, rotated 180°, and merged with the first to generate an antiparallel alignment setup with comparable water content in the two membrane-delineated compartments. A small offset (0.08 nm) was included to ensure no collapse at the edges of the two simulation boxes. A total of 20 ns of extra equilibration was included after energy minimization of the new double-bilayer simulation box. The ion-permeation pathway was defined by two 1.2-nm-radius cylinders centered at residue 247 (defined as the compartment boundary) extending 7.5 nm toward the channel ECD and 5 nm toward the channel ICD. The swapping frequency was set to 100, the threshold to 1, and the coupl-steps to 10. A comprehensive system-setup script and the corresponding mdp file (59) are available as indicated in *Data, Materials, and Software Availability*.

Potential differences were generated by varying sodium-ion concentrations in the aqueous compartments, keeping the chloride-ion concentration constant. Each simulation was run for 140 ns with protein Cα atoms restrained to prevent deviations from the starting state. The potential was quantified with the GROMACS potential tool in double precision. Net-zero charge of groups was assumed to improve accuracy. To reduce errors from limited precision during the double integration of charges, the calculation was performed in double precision while using options to force the net potential difference across the system to be zero, and the z-center of the system was translated to the origin to avoid asymmetric rounding errors. To calculate single-channel conductance, ionic current as a function of the potential difference was determined within 20-ns time windows, with 10-ns overlap between consecutive windows.

For electric-field simulations, a potential of ±200 mV was applied to the single-bilayer system. Modified channels without ECD, without ICD, or with the mutation E97A were embedded into the bilayer and equilibrated as described. The conductance was quantified by the corresponding current—i.e., the number of ions permeating during the simulations—times its valence, and then divided by the applied potential.

### AWH.

AWH methods have been widely applied to study the ion-permeation free-energy profiles in other channels (31, 32, 63). Unlike umbrella sampling, AWH does not have defined initial configurations, but flattens free-energy barriers along the reaction coordinate to converge to a freely diffusing ion. This method was used to calculate free-energy profiles along the pore axis for Na^+^, K^+^, Ca^2+^, and Cl^−^. For each equilibrated cryo-EM structure, one ion was additionally placed in the center of the pore around E258; a flat-bottomed restraint was applied to the ion to keep a radial distance below 20 Å from the pore axis. An independent AWH bias with a force constant of 12,800 kJ/mol/nm^2^ was applied to the center-of-mass z-distance between the selected ion and residue 247, with a sample interval across more than 95% of the box length along the *z* axis to reach periodicity. Semi-isotropic pressure coupling was used to keep the pressure to 1 bar, where the compressibility along the *z* axis was set to 0 to ensure a constant sampling coordinate. A total of 16 walkers sharing bias data and contributing to the same target distribution were simulated for >100 ns until the potential of mean force (PMF) profile converged in more than 10 ns.

### Coarse-Grained Simulations.

Coordinates for the same three α7-receptor cryo-EM structures (PDB ID codes 7KOO, 7KOX, and 7KOQ) without ions, ligands, or glycans were coarse-grained, through the representation of roughly four heavy atoms as a single bead, using Martini Bilayer Maker (64) in CHARMM-GUI (54). The protein was embedded in a symmetric membrane containing 20% CHOL, 16% POPC, 24% PIPC, 4% POPE, 12% PIPE, 4% POP2, 12% PIPI, 4% POPA, and 4% PIPA, which approximates the soy–lipid mixture used for experimental reconstitution of this receptor (16) (PC, phosphatidylcholine; PE, phosphatidylethanolamine; P2, phosphatidylinositol bisphosphate; P,: phosphatidylinositol; PA, phosphatidic acid; PO corresponds to a C16:0/18:1 lipid tail, while PI corresponds to a C16:0/18:2 lipid tail). In total, 2,500 lipids were inserted in a 27-nm × 27-nm × 20-nm simulation box, constituting ∼130,000 total beads, including water and ions. After energy minimization and equilibration in CHARMM-GUI, simulations were run with the protein restrained for 20 μs in GROMACS 2020 to allow lipid convergence, using Martini 2.2 and 2.0 parameters for amino acids and lipids (37), respectively.

The penalty of membrane compression was quantified (65) as

$${\text{G}}_{\text{compression}}\;=\;\frac{{\text{K}}_{\text{A}}}{2}\int^{\;}{\left(\frac{\text{u}\left(\text{r}\right)}{\text{l}}\right)}^{2}{\text{d}}^{2}\text{r}\text{,}$$

where *K_A_* is the bilayer area stretch modulus (∼60 kT/nm^2^), *u*(*r*) is the deformation from the unperturbed leaflet thickness, and *l* is the unperturbed leaflet thickness.

By summing the grid-based average membrane-thickness penalty,

$${\text{G}}_{\text{compression}}\;=\;\frac{{\text{K}}_{\text{A}}}{2}\underset{\mathrm{grid}}{\sum }{\left(\frac{u{\left(r\right)}_{\mathrm{grid}}}{l}\right)}^{2}S_{\mathrm{grid}},$$

the free-energy difference from the membrane-compression penalty can be calculated between three functional states:

$$\begin{array}{l} \Delta {\text{G}}_{\text{closed}\to \text{open}}=1.2\;kT\\ \Delta {\text{G}}_{\text{open}\to \text{desensitized}}=-0.7\;kT. \end{array}$$

### Simulations with PNU.

The coarse-grained model of PNU was built by using the CG builder tool (https://jbarnoud.github.io/cgbuilder/). CG bead types and bonded parameters were assigned according to the Martini 3 forcefield (39). Parameters were then optimized with Swarm-CG (66) to fit the all-atom parameters. Permeation PMFs along the POPC membrane bilayer were then profiled with umbrella sampling (described below) for validation. After converting the entire system to Martini 3, 10 PNU molecules were placed randomly into the simulation box (1 nm away from the protein). The backbone beads of the protein were restrained for better sampling. Four replicates of each system were then run for 20 μs each.

### Umbrella Sampling.

An output frame from coarse-grained simulations, in which one PNU was bound to the intersubunit binding site, was used as an initial configuration and backmapped to atomistic coordinates. The center-of-mass *x*/*y*-plane distance of PNU from the five position-253 residues, which would be in the middle of the pore, was used as the pulling coordinate. The “distance” was set to be the pulling-coordinate geometry. PNU was pulled either into the pore or out to the membrane at a rate of 0.0005 nm/ps with a force constant of 10,000 kJ/mol/nm^2^ to generate initial configurations, with an interval of 0.04 nm, spanning 0 nm to 3.7 nm. In total, 97 umbrella-sampling windows were simulated for 100 ns. The PNU was position-restrained with a flat-bottomed potential to keep it in a 2-nm layer parallel to the *x*/*y* plane for convergence. The weighted histogram analysis method (67) was used to analyze the results. To measure permeation of PNU across the POPC bilayer, a similar setup was applied to either the all-atom system in CHARMM36M or the coarse-grained system in Martini 3. Umbrella-sampling windows with an interval of 0.05 nm were generated, where the center-of-mass *z* distance between the PNU and the membrane was pulled with a “direction” geometry. The PBC atom of the membrane was set to −1 to turn on cosine weighting.

### Visualization and Analysis Tools.

Visualizations were created in VMD (68); most analyses were performed with GROMACS and MDAnalysis (69) and plotted with RainCloudPlot (70) and Matplotlib (71). For pore-radius calculation and visualization, CHAP (72) was used. G_elpot (73) was used for quantifying the electrostatic potential along the channel with or without Ca^2+^. For the coarse-grained simulations, MemSurfer (74) was used to quantify membrane thickness, and PyLipID (75) was used to measure and map the occupancy and residence time of different lipids onto the protein.

## Supplementary Material

Supplementary File

## Data Availability

Modified forcefield parameters, system-setup scripts, and simulation parameter files are available in Zenodo (DOI: 10.5281/zenodo.6998046)(76).
