# Supplementary material for: Differential interactions of resting, activated, and desensitized states of the α7 nicotinic acetylcholine receptor with lipidic modulators
Source: Proc Natl Acad Sci U S A. 2022 Oct 17;119(43):e2208081119. doi: 10.1073/pnas.2208081119 (PMC9618078; doi:10.1073/pnas.2208081119)
Supplement: Supplementary File [file pnas.2208081119.sapp.pdf]

Supplementary Information for

Differential interactions of resting, activated, and desensitized states of the α7 nicotinic acetylcholine receptor with lipidic modulators

Yuxuan Zhuang,[1] Colleen M Noviello,[2] Ryan E Hibbs,[2] Rebecca J Howard,[1] Erik Lindahl[1,3,*]

This PDF file includes:

Figures S1 to S14, S16

Video S15

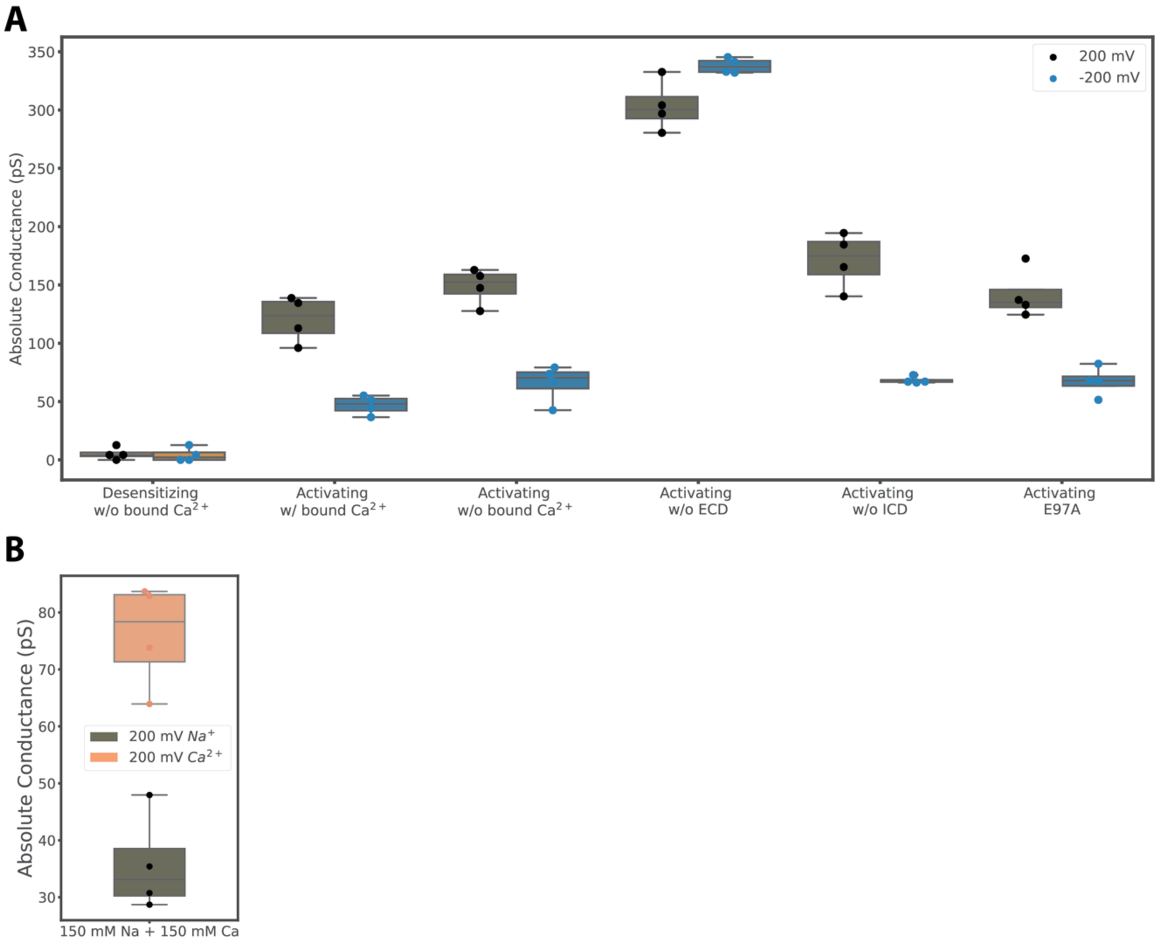

Figure S1. Applied electric field simulations support a conducting functional state in activating conditions. A. Average Na^+ conductance measured at 200 mV (gray) and -200 mV (blue) potentials for structures determined under (left–right) desensitizing conditions, activating conditions (with or without bound Ca^{2+}), activating conditions without the extracellular domain, activating conditions without the intracellular domain, or activating conditions with mutation E97A inside 150 mM NaCl solution. B. Average Na^+ and Ca^{2+} conductance measured at 200 mV potential for the structure determined under activating conditions inside 150 mM NaCl + 150 mM $CaCl_2$ solution.

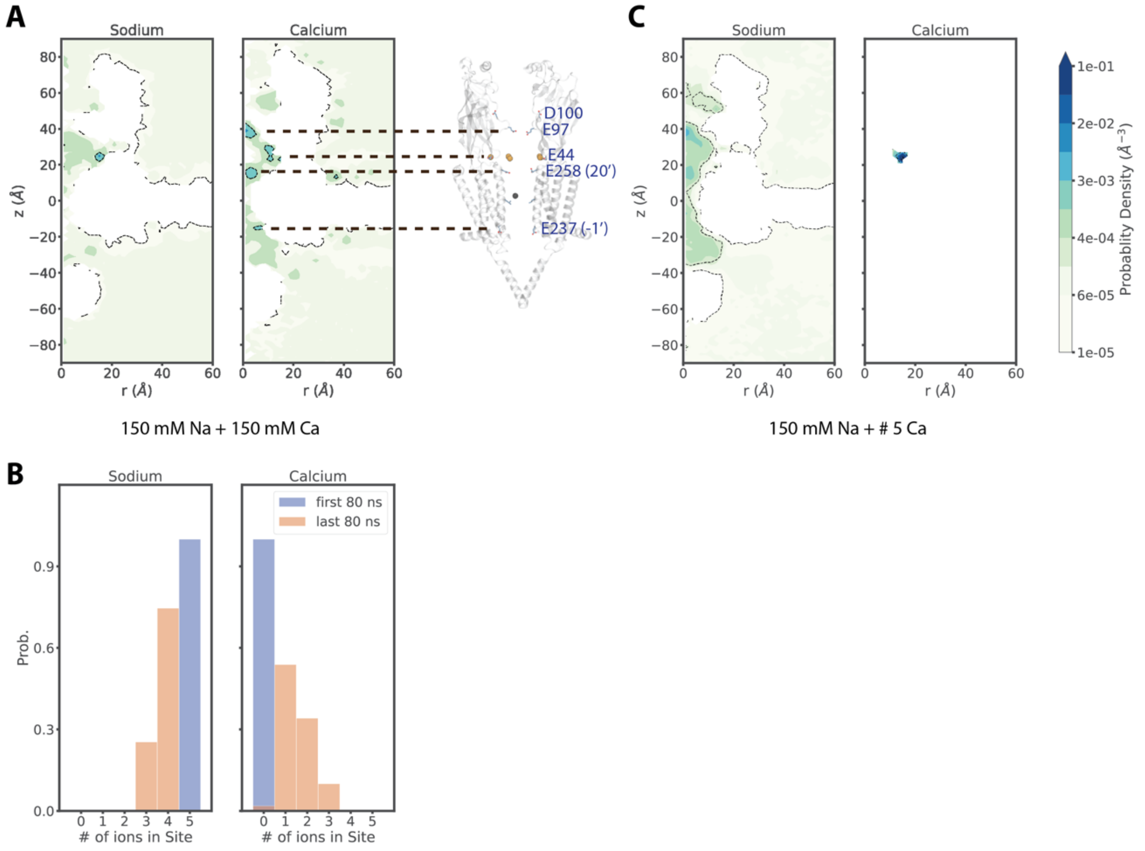

Figure S2. Ion density of Na^+ and Ca^{2+} in applied electric field simulations. A. Ion density of Na^+ (left) and Ca^{2+} (right) for the structure determined under activating conditions. After equilibration in 150 mM NaCl and no Ca2+, the system was simulated at 200 mV in 150 mM NaCl + 150 mM $CaCl_2$. The density was calculated for the last 80 ns of simulation time, and plotted on a two-dimensional plane defined by the distance along the pore axis (z) and radial distance from the pore axis (r). The structurally resolved Ca^{2+} binding site near E44 could host both Na^+ and Ca^{2+}. **B.** Probability distributions for Na^+ and Ca^{2+} ions in five E44 sites during the first (blue) or last (orange) 80 ns of simulation in 150 mM NaCl + 150 mM $CaCl_2$. The Ca^{2+} ions were placed freely inside the bulk solvent at the beginning of the simulation, but equilibrated over time to occupy 1–3 protein sites, displacing Na+ ions. **C.** Ion density of Na^+ (left) and Ca^{2+} (right) for the structure determined under activating conditions, simulated in 150 mM NaCl solution + 5 bound Ca^{2+}. The density was calculated for the last 80 ns simulation time, and plotted as in panel A. The Ca^{2+} remained bound in its structurally resolved sites, precluding prominent occupancy by Na+. In panels A and C, ion density is indicated (white–green–blue) according to scalebar at far right. Note area of zero density corresponding to the ICD (z < -40 Å), indicating a nonlinear conduction pathway below the transmembrane pore.

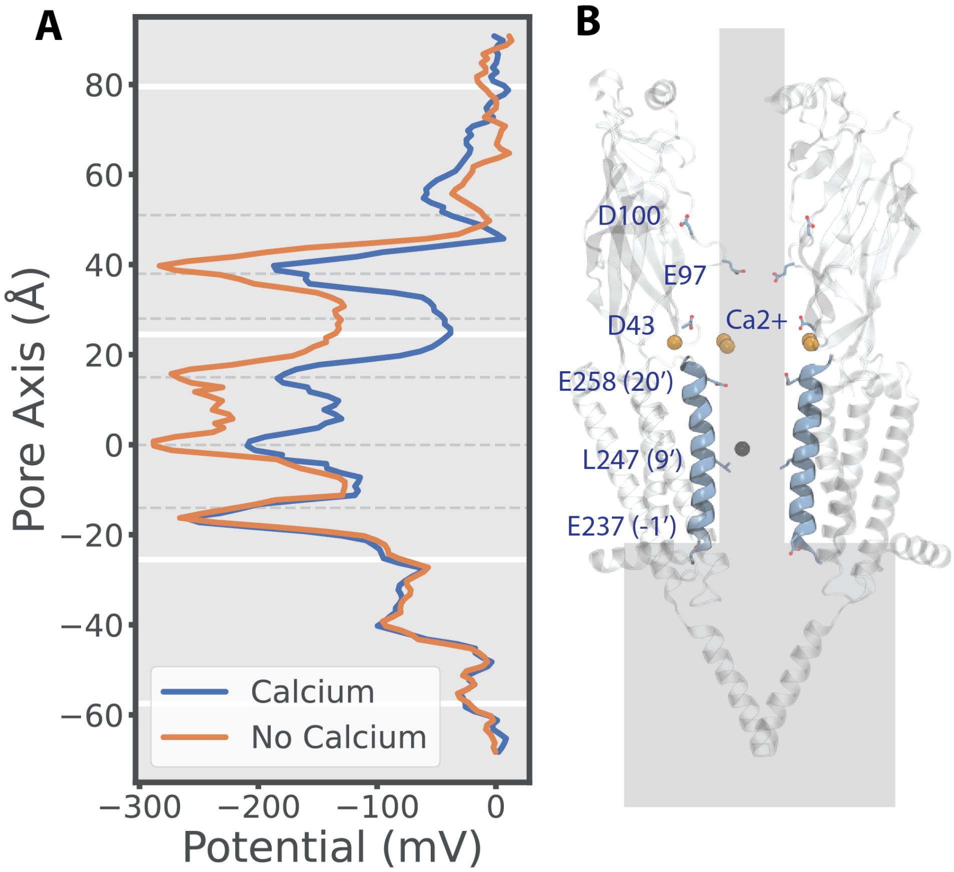

Figure S3. Electrostatics along the channel pore. A. The electrostatics along the channel with or without Ca^{2+} calculated with g_elpot. **B.** The electrostatics calculated region mapped onto the structure under activating conditions. Key residues are shown as sticks, along with five Ca^{2+} ions (ochre) resolved in the ECD.

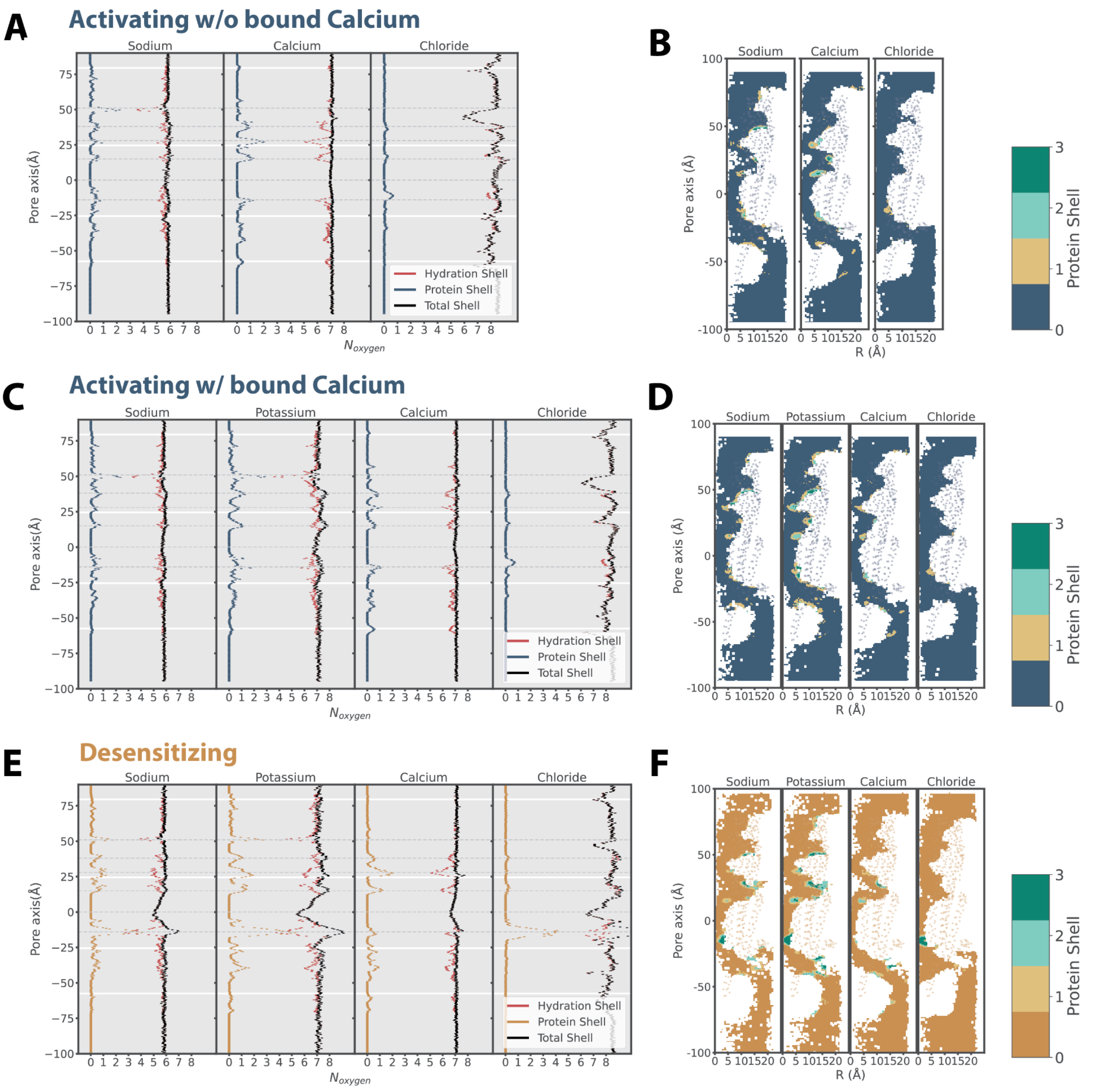

Figure S4. Water/protein coordination for various ions. A. The average number of coordinated oxygen atoms from water/protein as a function of position along the pore axis in the activated state without bound Ca^{2+}. **B.** The heat map of the average number of coordinated oxygen atoms from protein in the activated state without bound Ca^{2+}. **C.** The average number of coordinated oxygen atoms from water/protein as a function of position along the pore axis in the activated state with bound Ca^{2+}. **D.** The heat map of the average number of coordinated oxygen atoms from protein in the activated state with bound Ca^{2+}. **E.** The number of coordinated oxygen atoms from water/protein as a function of position along the pore axis in the desensitized state. **F.** The heat map of the average number of coordinated oxygen atoms from protein in the desensitized state.

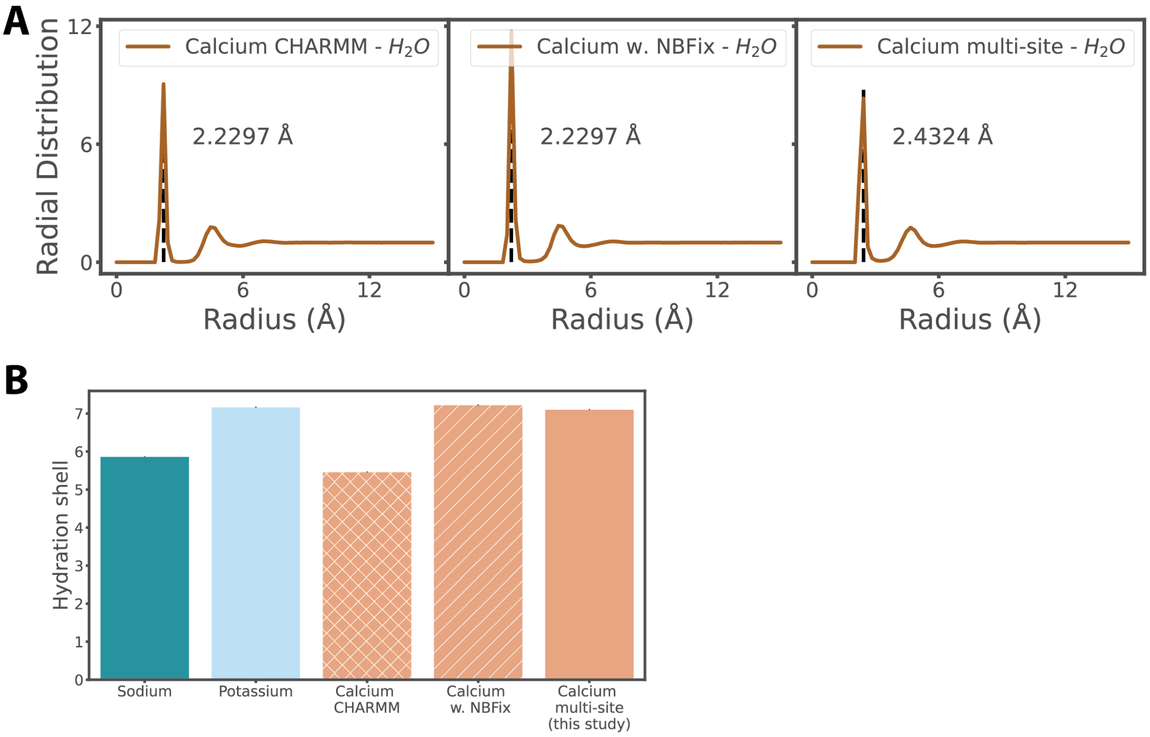

Figure S5. Comparative performance of Ca^{2+} models. A. Radial distribution function of ion-water oxygen atom pairs using the (left–right) CHARMM36, revised CHARMM36 with NBFix, and multi-site (CAM) Ca^{2+} models. **B.** Hydration shells of various ion species.

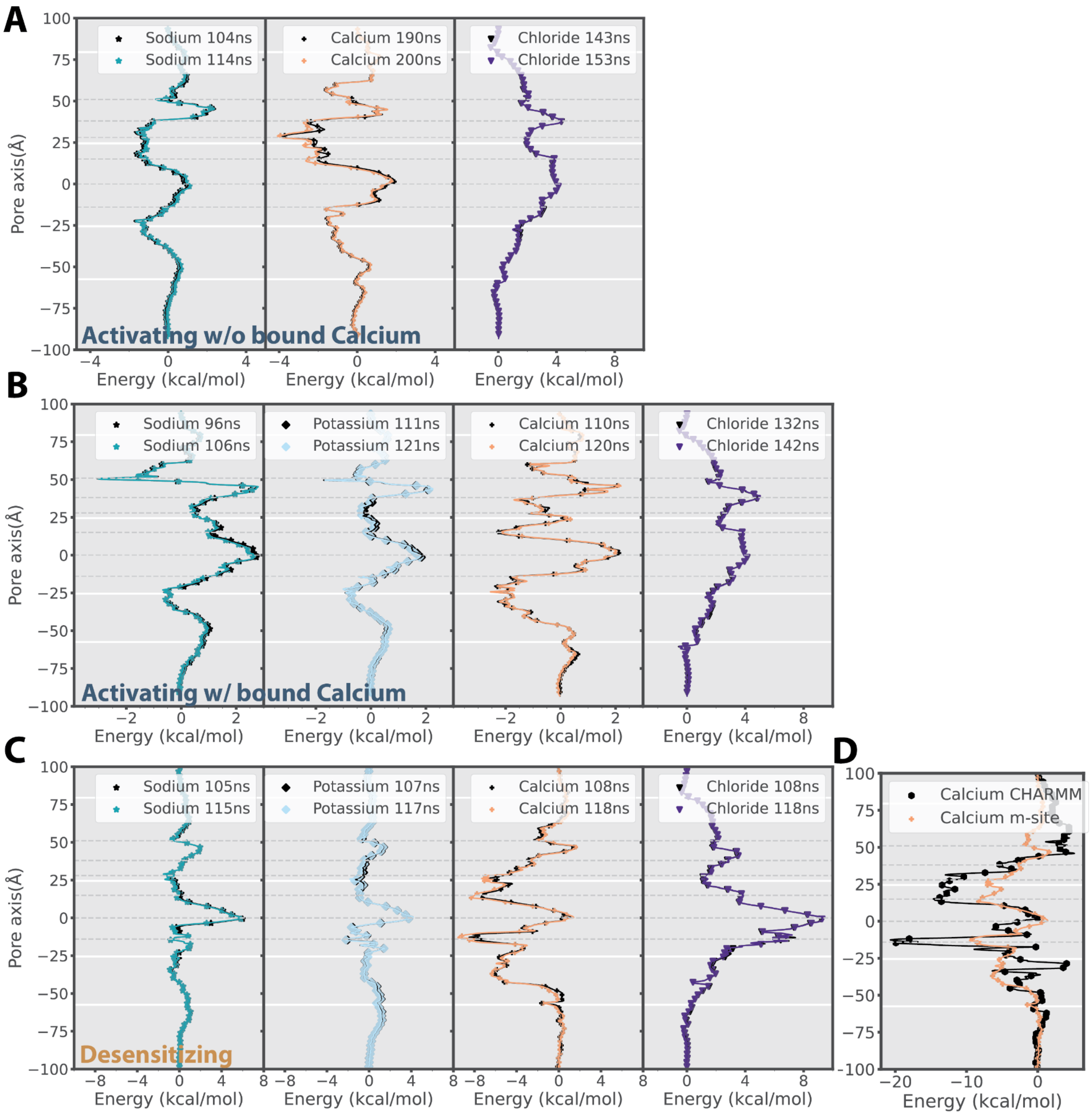

Figure S6. Convergence of permeation free-energy profiles. A. The free energy profiles of ion permeation for the activated state without bound Ca^{2+}. **B.** The free energy profiles of ion permeation for the activated state with bound Ca^{2+}. **C.** The free energy profiles of ion permeation for the desensitized state. **D.** Comparing the free energy profiles of Ca^{2+} permeation for the desensitized state with the CHARMM36 parameter and the revised multi-site Ca^{2+} model (CAM).

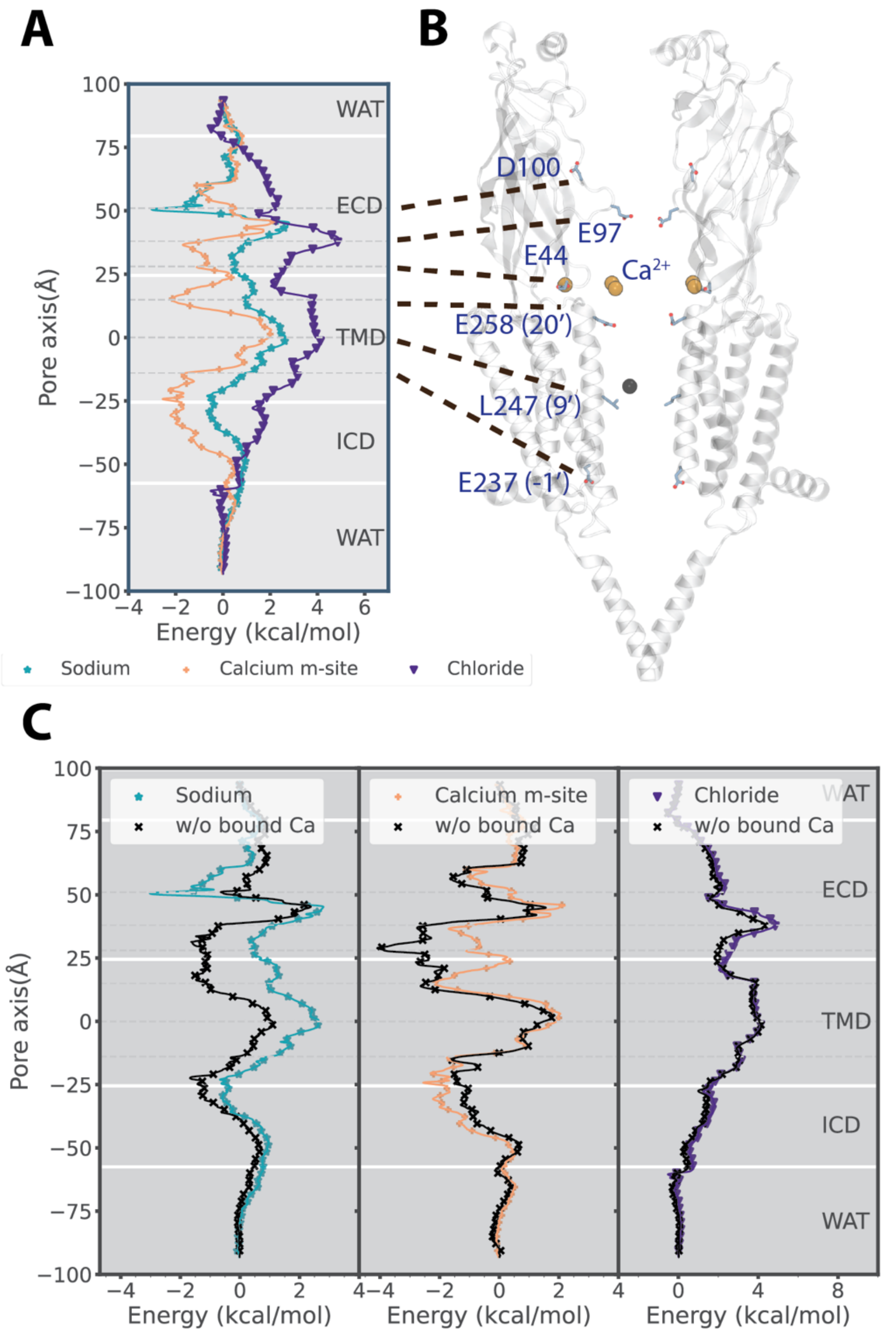

Figure S7. Bound Ca^{2+} perturbs permeation free-energy profiles in the activated state. A. Free-energy profiles of Na^{+} (teal), Ca^{2+} (ochre), and Cl^{-} (indigo) permeation through the structure determined under activating conditions (PDB ID 7KOX) with resolved Ca^{2+} ions explicitly included in simulations. **B.** Protein model of the structure determined under activating conditions; for clarity, only two opposing subunits are shown. Key residues labeled in panel A are shown as sticks, along with five Ca^{2+} ions (ochre) resolved in the ECD. **C.** Comparison of free-energy profiles with or without bound Ca^{2+} for the structure determined under activating conditions.

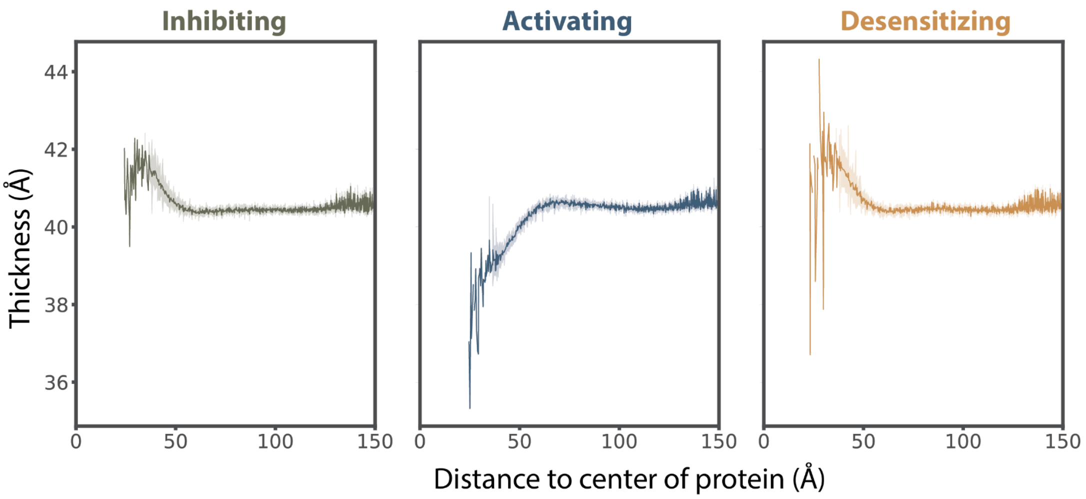

Figure S8. Quantification of membrane thickness. The perturbed membrane thickness differences averaged over the last 5 μs simulations dissipated within 60 Å from the protein for the lipid-embedded α7-nAChR structure under inhibiting (resting, left), activating (activated, center), or desensitizing (desensitized, right) conditions.

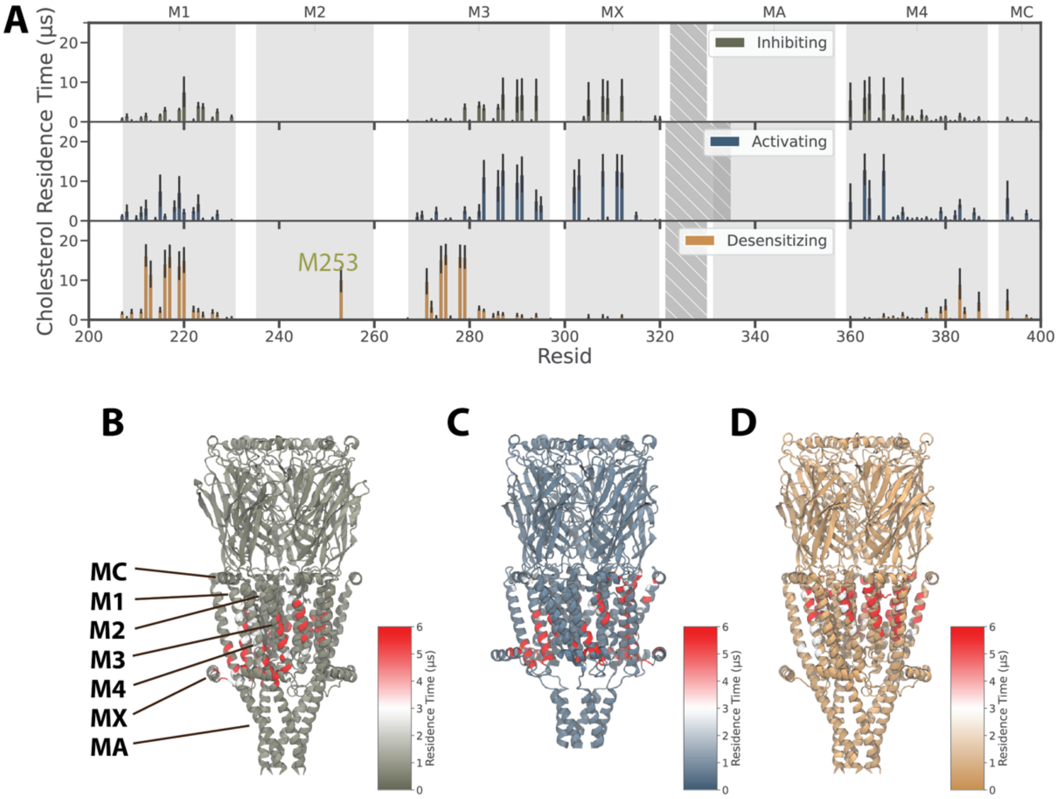

Figure S9. Residence time of cholesterol interactions in the desensitized state. A. The residence time of cholesterol interactions with each residue of the α7 nAChR in 20-us coarse-grained simulations of structures determined under inhibiting (top), activating (middle), and desensitizing conditions (bottom). While the resting and activated states shared a similar cholesterol interaction site in the lower part of the transmembrane domain the desensitized state shifted the cholesterol interactions upwards. **B.** Cholesterol residence time as in *A,* colored according to scalebar and mapped onto the experimental structure under inhibiting conditions, with key membrane-facing or peripheral helices labeled. **C.** Cholesterol residence time as in *B* for the structure under activating conditions. **D.** Cholesterol residence time as in *B* for the structure under desensitizing conditions.

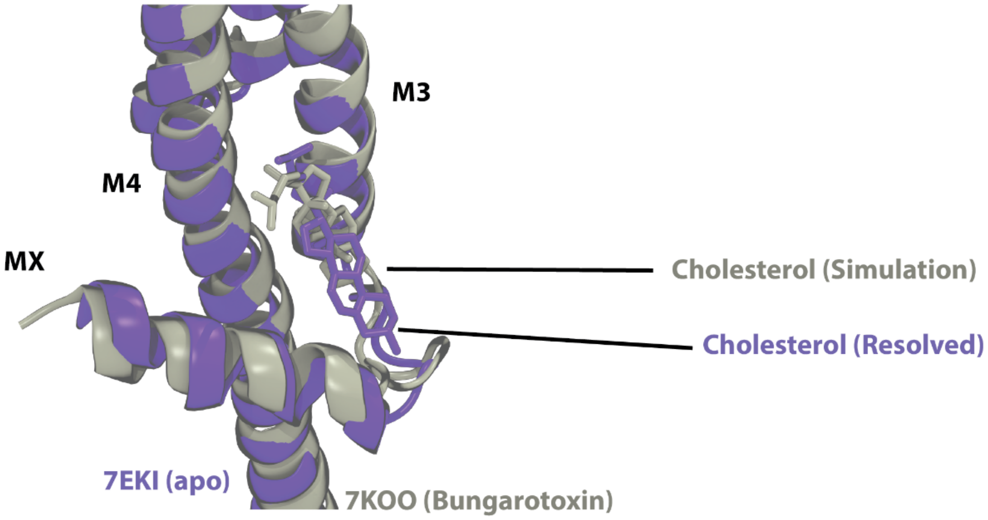

Figure S10. Cholesterol sites. Comparison of the predicted cholesterol binding site in the a-bungarotoxin structural model (gray, PDB: 7KOO) and the resolved cholesterol binding site in the apo structural model (purple, PDB: 7EKI).

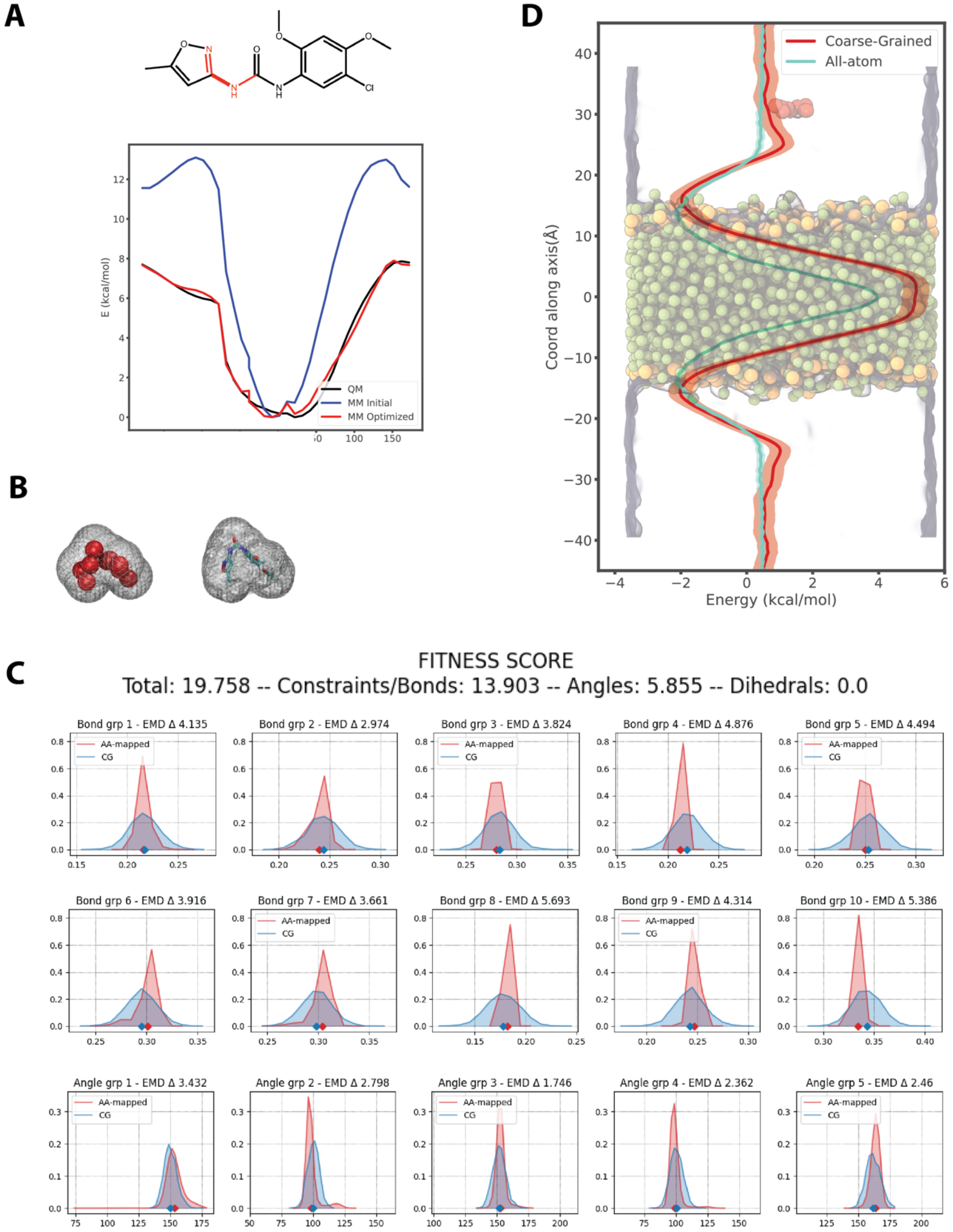

Figure S11. Optimization and validation of PNU parameters. A. Optimized energy landscape of one torsion angle parameter of the atomistic PNU. **B.** Comparison of solvent accessible surface area (SASA) between atomistic and coarse-grained PNU. **C.** Bonded term optimization and scoring by SWARM-CG for PNU. **D.** Comparison of bilayer (POPC) permeation free energy of atomistic and coarse-grained PNU.

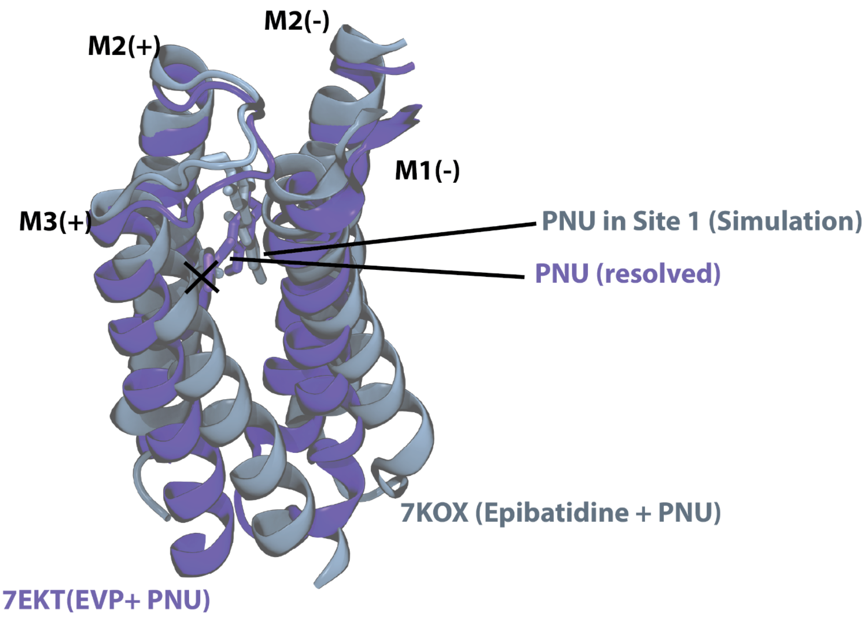

Figure S12. PNU sites. Comparison of predicted PNU binding site in Epibatidine+PNU structural model (gray, PDB: 7KOX) and resolved PNU binding site in EVP+PNU structural model (purple, PDB: 7EKT). The predicted PNU bound with its long axis parallel to the membrane plane while the experimentally resolved PNU had this axis oriented perpendicularly.

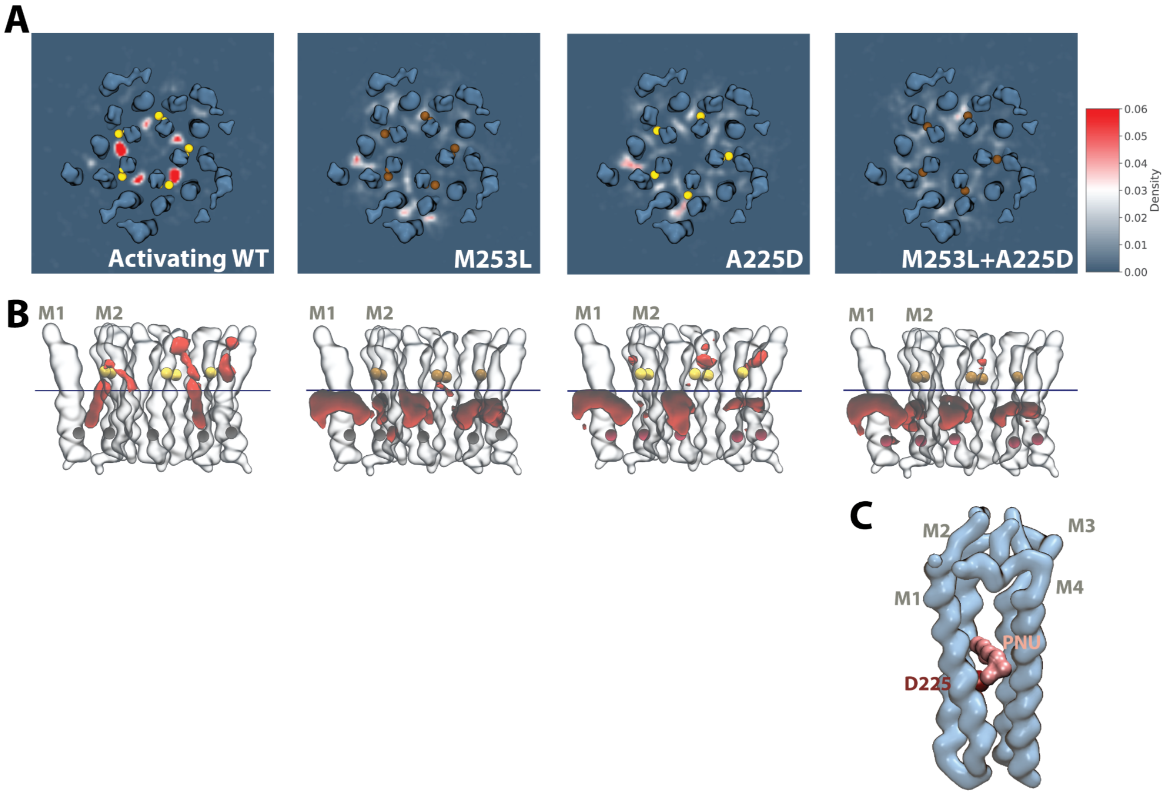

Figure S13. Simulations of mutant systems confirm the PNU binding sites. A. The PNU density map derived from 20 * 4 μs simulations in different mutant systems. **B.** The corresponding PNU density (red) in different mutant systems. The slice shown in A-D was plotted as a blue horizontal line. The residue 253 was shown as either yellow (M) or brown (L) bead; The residue 225 was shown as either grey (A) or ruby (D) bead. Only M1, M2 were shown as transparent surfaces. **C.** A snapshot from the CG simulations showed the possible binding mode of PNU (pink) in the mutant system including interaction with D225 (dark red).

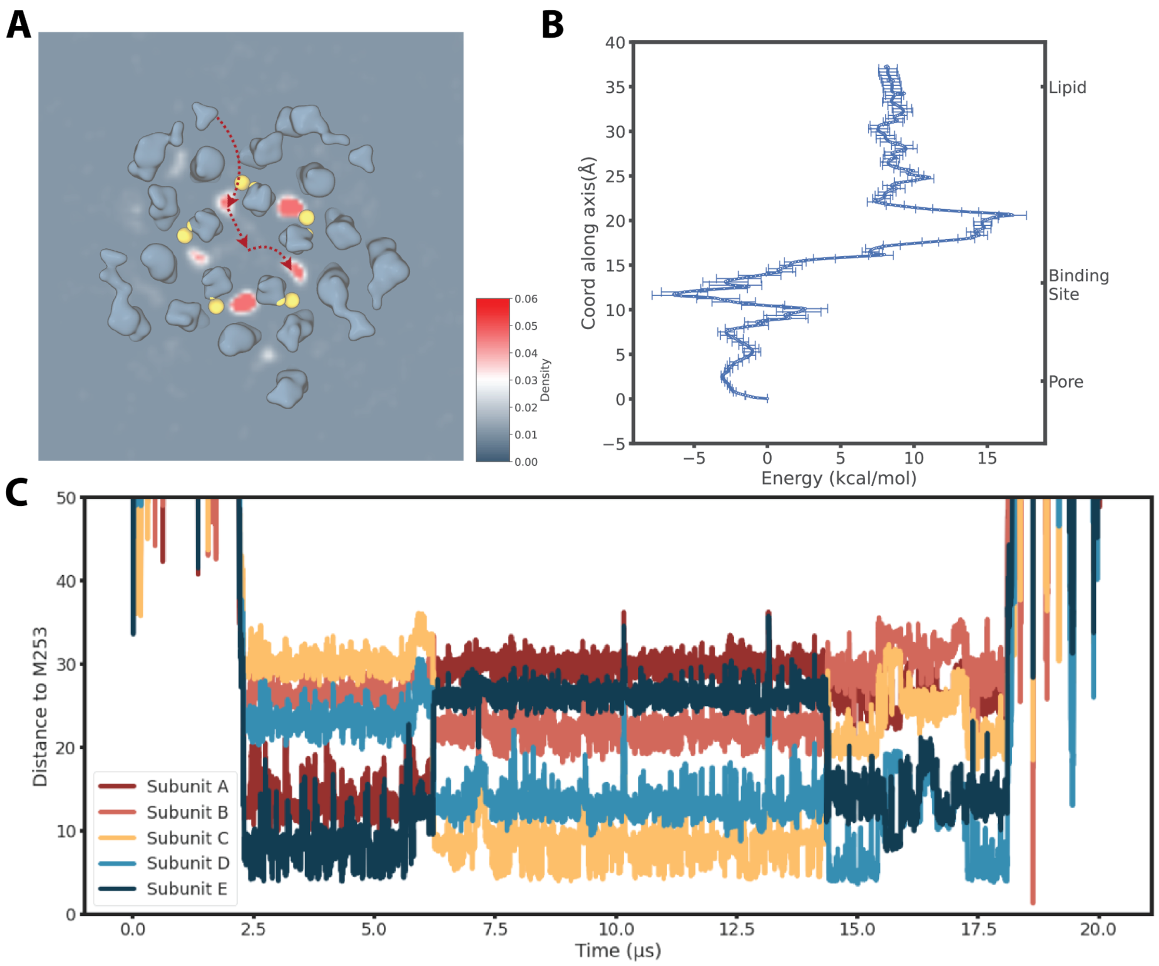

Figure S14. Spontaneous PNU transits between site-1 interfaces in coarse-grained simulations. A. Illustration of a PNU molecule diffused from site 1 in one of the subunits across the pore into site 1 located in another subunit in CG simulations. **B.** Free energy landscape of PNU transiting from the pore into the membrane via site 1 by atomistic umbrella sampling simulations. **C.** The time evolution of the distance of one PNU molecule with five M253 in one CG simulation. Aside from entering/exiting the site 1 from/into the bilayer region at ~2 μs and ~18 μs. The switching of binding sites can be visualized at ~6 μs, and ~14 μs.

Video S15. Movie of spontaneous PNU transition between site-1 interfaces in coarse-grained simulations.

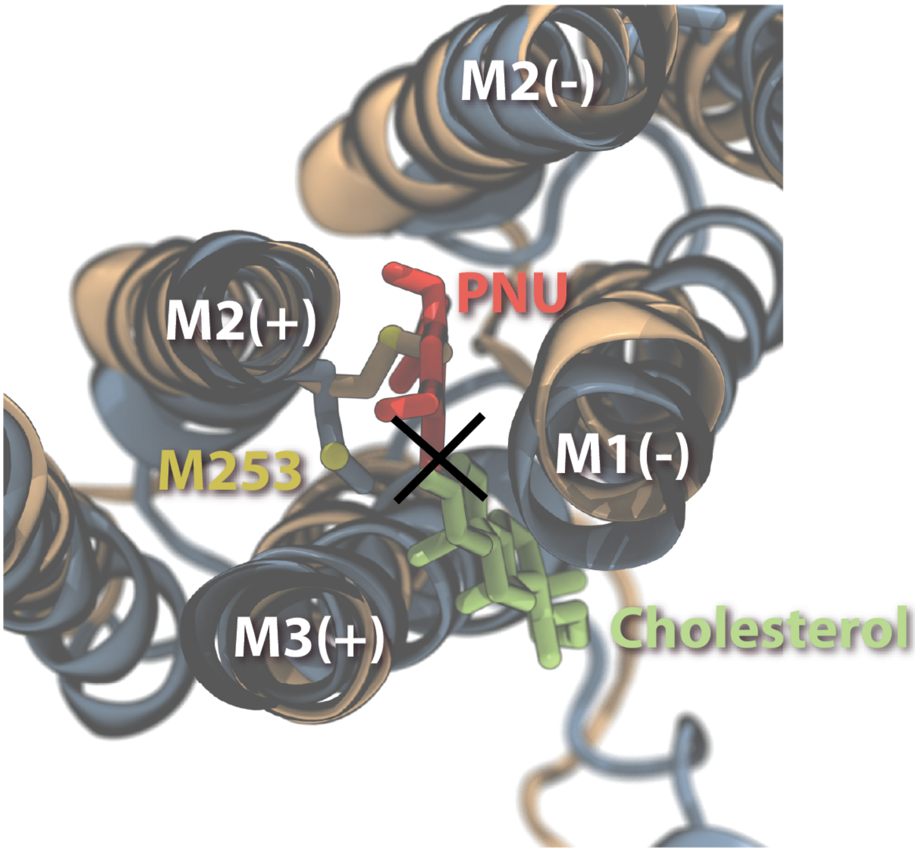

Figure S16. State-dependent interfacial binding. Superposition of the proposed cholesterol binding site in the desensitized state and the proposed PNU binding site in the activated state.
